# XPA confers ability of endonucleases to act processively and to incise damaged nucleosomal DNA

**DOI:** 10.3389/ebm.2026.10902

**Published:** 2026-08-11

**Authors:** Muriel W. Lambert, W. Clark Lambert

**Affiliations:** Department of Pathology, Immunology and Laboratory Medicine, Rutgers New Jersey Medical School, Newark, NJ, United States

**Keywords:** DNA endonucleases, DNA repair, nucleosomal DNA, protein processivity, xeroderma pigmentosum, XPA gene, XPA protein, chromatin-remodeling

## Abstract

Repair of damaged DNA is a complex process, particularly when it is compacted into nucleosomes. There are a number of genetic disorders with deficiencies in DNA repair. Knowledge of the genes and proteins involved in these repair deficiencies is critical in developing an understanding of the molecular mechanisms utilized by proteins in the DNA repair pathways. One of these genetic disorders is xeroderma pigmentosum (XP), which is defective in nucleotide excision repair (NER). Patients in XP complementation group A (XP-A) are among the most severely affected with the lowest levels of DNA repair. The XPA protein, which is defective in these patients, plays a number of roles in the DNA repair process. One particularly important role proposed is acting as a processivity factor enabling endonucleases (XPF and XPG) and the XPB/TFIIH translocase to localize to damage sites using a processive mechanism of action. Another proposed role is in interacting with chromatin-remodeling proteins so as to enhance accessibility of lesions in nucleosomal DNA to endonucleolytic incision and other DNA repair activities. In XP-A cells, the XPA protein is proposed to be defective in ability to act as a processivity factor; endonucleases localize damage sites by a distributive mechanism and are also defective in incision of damaged nucleosomal DNA. This defect is corrected by recombinant normal human XPA. Mutations in exons 3 and 5 in the DNA binding domain of the *XPA* gene lead to loss of ability of XPA to act as a processivity factor. The mutation in exon 5 was found in two XP-A patients with severe XP. These studies emphasize the importance of correlating specific mutations in an XP gene and the resulting defect in a particular repair protein with the clinical severity of XP and could lead to development of novel therapeutic approaches for this disorder.

## Impact statement

Xeroderma pigmentosum (XP) is a genetic disorder which is defective in nucleotide excision repair (NER). This review concentrates on XP complementation group A (XP-A) and emphasizes two important roles of the XPA protein in DNA repair. It is proposed to enhance a processive mechanism of action in DNA endonucleases (XPF and XPG) and the XPB/TFIIH translocase and to interact with chromatin-remodeling proteins to increase accessibility of lesions in nucleosomal DNA. In XP-A cells loss in ability of XPA to act as a processivity factor results in decreased endonuclease processivity and decreased endonucleolytic incisions on damaged nucleosomal DNA. Mutations in the DNA binding domain of the XPA gene lead to this defect and correlate with clinical severity of XP. Such correlations between a specific mutation in a repair gene and a functional defect in a DNA repair protein with the clinical manifestations of XP are centrally important in designing NER-targeted therapeutics.

## Introduction

Xeroderma pigmentosum (XP) is a rare, genetically transmitted disorder characterized by extreme sensitivity to sunlight and ultraviolet irradiation (UV) induced pigmentary changes in the skin and an elevated incidence of skin cancers [1–10]. It has a worldwide prevalence of 1:1,000,000. This is a heterogenous disorder. The underlying genetic defects are the result of mutations in nine genes. Eight of the XP genes are assigned to complementation groups, XP-A through XPG and XP-J [2–12]. Complementation groups XP-H and XP-I have been withdrawn since they were found to be identical to XP-D and XP-C, respectively [11]. The XP genes are associated with defects in DNA nucleotide excision repair (NER) [2–12]. The ninth group, the XP variant (XPV), has mutations in the *POLH* gene, and is deficient in lesion bypass during DNA replication (translesion DNA synthesis) [3–6, 13–18]. XP patients clinically show skin dryness, also called xerosis, hence the term “xeroderma” and progressive pigmentary abnormalities with freckle-like pigmentation, hence the term “pigmentosum” [2–8]. They have different levels of skin damage and early photoaging of the skin. In some cases, neurological abnormalities of varying severity occur [2–8, 19]. UV induced damage to both the skin and eyes can lead to development of multiple malignant tumors [1–9]. No curative treatment is available for XP patients at the present time. Life-long avoidance to sun exposure and protection from it is thus critical. A number of therapeutic approaches have been and are being used to help prevent or delay the clinical manifestations of the disorder after UV exposure [20–24]. Molecular analysis of the specific XP gene mutations and the resulting defective DNA repair proteins in XP can help in determining how these defects affect the functioning of the XP proteins and will aid in understanding the molecular mechanisms involved in their role in DNA repair and in the clinical manifestations and progression of this disorder.

Patients in the XP-A complementation group are some of the most severely affected and have the lowest levels of DNA repair [2–8]. The XPA protein, encoded by the *XPA* gene, is a critical component of the NER pathway; it has multiple functions and is a significant coordinator of NER dynamics [25–29]. It is examined in more detail in this review. In particular, the important role XPA is proposed to play in influencing the mechanism by which repair proteins localize to sites of damage during the repair process and the effect this has on the repair of damaged nuclear DNA when it is organized into nucleosomes is discussed. The mechanism a protein uses to translocate along DNA can have an important effect on the rate and efficiency of this process. Studies carried out by Lambert et. al. indicate that XPA, in normal human cells, aids in conferring a processive mechanism of action on endonucleases involved in NER (i.e., XPF and XPG) [25]. Studies by Kokic et al. and Kappenberger et al. indicate that XPA from the eukaryotic fungus, *Chaetomium thermophilum,* is involved in increasing the processivity of the XPB/TFIIH translocase as it progresses along UV-damaged DNA [30, 31]. These studies together suggest that XPA may have an important role in stimulating a processive mechanism of action on several proteins involved in the NER process, which could potentially markedly enhance the efficiency of the repair process.

Since DNA in the nucleus wraps around histones, forming nucleosomes, knowledge of the effect nucleosome structure can have on repair protein activity is important and highly relevant to the clinical manifestations of the disorder. It has been proposed that a processive mechanism of action of endonucleases is important for the increased production of incisions observed on damaged nucleosomal DNA in normal human cells [25, 32]. Chromatin-remodeling proteins are also hypothesized to be involved in this process and increase the accessibility of sites of DNA damage to DNA repair proteins. In contrast, in several XP-A cell lines, mutations in the *XPA* gene lead to production of an XPA protein which does not act as a processivity factor; endonucleases involved in DNA repair in these XP-A cells localize to sites of damage by a distributive rather than a processive mechanism of action and are defective in ability to incise UV-damaged nucleosomal DNA [25, 32]. These studies thus suggest that the mechanism an endonuclease uses to translocate along DNA to sites of damage (i.e., whether it is processive or distributive) can be important in determining its ability to incise damaged DNA when it is present in nucleosomes and that chromatin-remodeling proteins play an important role in this process. These studies also indicate that mutations in the DNA binding domain in the *XPA* gene, lead to loss of ability of XPA to enhance the processivity of endonucleases, and to decreased endonucleolytic incision at sites of damaged nucleosomal DNA [33]. This in turn could potentially be related to the increased severity of this disorder in these patients [33]. Understanding the molecular basis of the disease-associated mutations in *XPA* thus requires a complex understanding of XPA-DNA interactions, particularly in the context of protein interactions with damaged nucleosomal DNA. Knowledge of the specific proteins and their different roles in the repair process could lead to the development of therapeutic approaches for treatment of this disorder.

## Clinical characteristics of XP

One of the major clinical characteristics of XP is the extreme sensitivity to UV light exposure, the early xerosis and hyperpigmentation of sun exposed skin, and the development of UV light induced skin cancers and pre-cancers [2–10]. Though only about 60% of XP patients are extremely sensitive to UV light, all patients show increased freckle-like hyperpigmented macules on sun-exposed skin [2–10]. Malignant skin cancers (basal cell carcinoma, squamous cell carcinoma, melanoma) develop in 70% of XP patients with a median age of 8 years [2–10]. These cutaneous neoplasms have been attributed to a defect in ability to carry out NER of DNA damage produced by UV-irradiation in sun-exposed skin in patients in XP complementation groups XP-A through XP-J [3–12]. XP-V patients, are able to carry out NER, however, they are defective in ability to bypass UV-irradiation induced DNA lesions at the time of DNA replication which can lead to development of cutaneous neoplasms [13–18]. The severity of the skin lesions produced can depend upon the level of exposure to UV light and also upon the XP complementation group and on the location of the genetic mutation in the specific XP gene within that group [18, 26, 27, 33–35]. Regardless of the complementation group, lesions are most prevalent on the face, head and neck.

In addition to hyperpigmented lesions and cutaneous neoplasms, other pathological changes are present in XP patients. These include xerosis and poikiloderma on sun-exposed skin, ocular abnormalities and progressive neurologic abnormalities [2–12]. Neurologic abnormalities and progressive neurological degeneration are found in about 25% of XP patients and include patients in XP-A, XP-B, XP-D, XP-F and XP-G complementation groups [2–10, 19]. Involvement typically includes progressive hearing loss, progressive cognitive impairment, loss of ability to walk and difficulty swallowing [2–4, 10, 19, 36].

There are different levels of DNA repair, as determined by measuring levels of DNA repair related unscheduled DNA synthesis (UDS), in patients in each of the eight complementation groups and in XP-V patients (Table 1). UDS is the DNA synthesis that occurs after the DNA damage has been excised and is different from the DNA synthesis that occurs during normal cell replication. It is quantified by determining the number of nucleotides that are incorporated into the newly synthesized DNA that replaces the damaged section of DNA during DNA NER [4, 5]. Within each complementation group there can be variation, which may be attributed to pathogenic variants in a specific gene. [2, 4, 5, 8]. XP-A patients have some of the greatest disease severity and the lowest levels of DNA repair (UDS) [4, 5]. UDS is usually 2–5% of normal following UV-irradiation (Table 1) Among the eight complementation groups, the *XPA* gene is the one most affected and XP-A represents 30% of all XP patients, followed by XP-C with 27% and XP-D with 15% (Table 1) [3, 4, 8, 10]. Genes in complementation groups XP-B, XP-E, XP-F, and XP-G are the least frequently affected (Table 1) [3, 4, 8, 10]. Defects in the *POLH* gene in XPV patients make up 23.5% of all cases of XP and cells from these patients are not deficient in NER and show 100% UDS (Table 1) [3, 4, 8, 10].

**TABLE 1:** DNA repair capabilities of cells from patients with xeroderma pigmentosum.

Complementation Group	Defective gene	Frequency (%)	UDS following UVC irradiation (% of normal)
XP-A	*XPA*	30	2–5
XP-B	*XPB/ERCC3*	0.5	3–7
XP-C	*XPC*	27	10–20
XP-D	X*PD/ERCC2*	15	25–50
XP-E	*XPE/DDB2*	1	40–50
XP-F	*XPF/ERCC4*	2	10–20
XP-G	*XPG/ERCC5*	1	2–25
Variant	*XPV/POLH*	23.5	100

Data from: Lambert and Lambert [4].

The greatest clinical severity of XP usually correlates with patients having the lowest levels of UDS, however, this is not always the case. It has been found that an XP patient may have low levels of UDS, and thus low levels of DNA repair, and only mild clinical features of XP [7]. One example is seen in several XP-A patients who have a similar mutation in intron 4 of the *XPA* gene [7]. These patients have barely detectable UDS but have mild clinical features of XP and no neurological problems [7]. However, 5% of the normal XPA protein was found in fibroblasts from these patient’s, which demonstrated that this low level of normal XPA protein was sufficient to alleviate the most severe clinical features of XP [7].

Studies are being carried out to further undertake a molecular analysis of the XP genes affected in different XP patients in order to try to determine the specific mutations which are responsible for the pathological changes observed in these patients. Since XP occurs via a loss-of function mechanism of any of the NER proteins or DNA polymerase η, elucidating the effect these mutations have on the function of specific repair proteins and their mechanism of action in the repair process is critical for developing a better understanding of the clinical abnormalities and neoplasia which result from exposure to DNA damage.

## Response of XP cells to DNA damage

Genomic DNA is continuously attacked by a plethora of damaging agents. A major source of exogenous DNA damage comes from UV-irradiation and this is the primary factor involved in the production of pigmentary abnormalities and neoplastic lesions characteristic of the skin of XP patients. Sunlight produces both long wavelength UVA (315–400 nm) irradiation and mid wavelength UVB (280–315 nm) radiation. Both wavelengths of UV-irradiation induce DNA damage in the skin epidermis, which includes mainly cyclobutene pyrimidine dimers (CPDs) and pyrimidine-pyrimidone (6-4) photoproducts ((6-4)PPs) [3, 4, 37]. The cited percentage of these lesions is 75% CPDs and 25% (6-4)PPs, though other minor forms of damage may be present [4, 38]. UVA also penetrates the dermis where it can additionally induce DNA lesions [3, 4]. Since all XP cells are defective in ability to either repair these forms of UV-induced DNA damage or accurately replicate past them, a large number of lesions form in the skin and eyes of these patients putting them at a high risk of developing skin tumors [2–10, 13–18]. For XP patients, groups XP-A through XP-J, failure to repair DNA damage by NER is thus a major underlying cause for this disorder [1–4, 6–8, 11, 12]. In XP variant patients, the inability to accurately replicate DNA past a site of damage is a major underlying cause [14–18, 39].

Cyclobutane pyrimidine dimers and the (6-4) photoproduct can be repaired by NER [2–5]. Other mechanisms of DNA repair may be involved in repair of some of the minor UV photoprocducts that form from UV irradiation. However, since the major photoproducts producted by UV irradiation are repaired by NER, which is defective in XP patients, this is the repair pathway which will be discussed. XP-A through XP-J patients are defective in ability to carry out NER of UV photodamage and other types of bulky adducts [2–5]. Each one of the XP genes in these patients is involved in NER and each is defective in a different step in this DNA repair pathway [1–8]. Loss of function of any one of the XP proteins leads to a defect in the entire NER repair pathway and to the pathological changes observed in XP patients. The NER pathway is highly coordinated. XP-V patients are able to carry out NER but are deficient in ability to bypass UV-induced lesions during DNA replication [10, 13–18, 35]. They are deficient in a specific DNA polymerase, pol η, a translesion DNA polymerase involved in inserting nucleotides in the newly synthesized DNA strand at the site of damage [10, 13–18, 35]. Defects in processing UV-induced photoproducts in both groups of XP patients lead to the clinical manifestations of the disorder. Examination of the gene defects in XP patients has shown the importance of these genes in the repair process. Thus, understanding the mechanism of action of each of the XP proteins in the repair pathway and their interactions with each other is critical in order to develop strategies to counteract the effects of their deficiency.

### NER defects in XP complementation groups XP-A through XP-J

NER is divided mechanistically into two sub-pathways: global genome NER (GG-NER) and transcription-coupled NER (TC-NER) [40–42]. GG-NER is involved in detecting and repairing a number of bulky or helix-destabilizing DNA lesions over the entire genome [42, 43]. TC-NER is responsible for the accelerated repair of lesions in the transcribed strand of DNA actively undergoing transcription [40–42]. These pathways differ in their mode of damage recognition, however, after the damage is recognized, the incision and repair synthesis steps are shared by both NER subpathways [40–43]. XP patients (XP-A through XP-J) are defective in GG-NER [1–8]. The newest XP complementation group, XP-J, contains biallelic mutations in the *GTF2H4* gene encoding the p52 subunit of TFIIH [11, 12]. Patients in each of these complementation groups are defective in a different step in the GG-NER process. This emphasizes that a defect in any one of the XP proteins involved in NER can lead to defects in the entire repair pathway and to the clinical manifestations of the disorder. The steps in normal GG- NER pathway are briefly described below. Since there is a high incidence of CPDs and (6-4)PPs in XP cells, this type of DNA damage will be used as an example.

**FIGURE 1:**
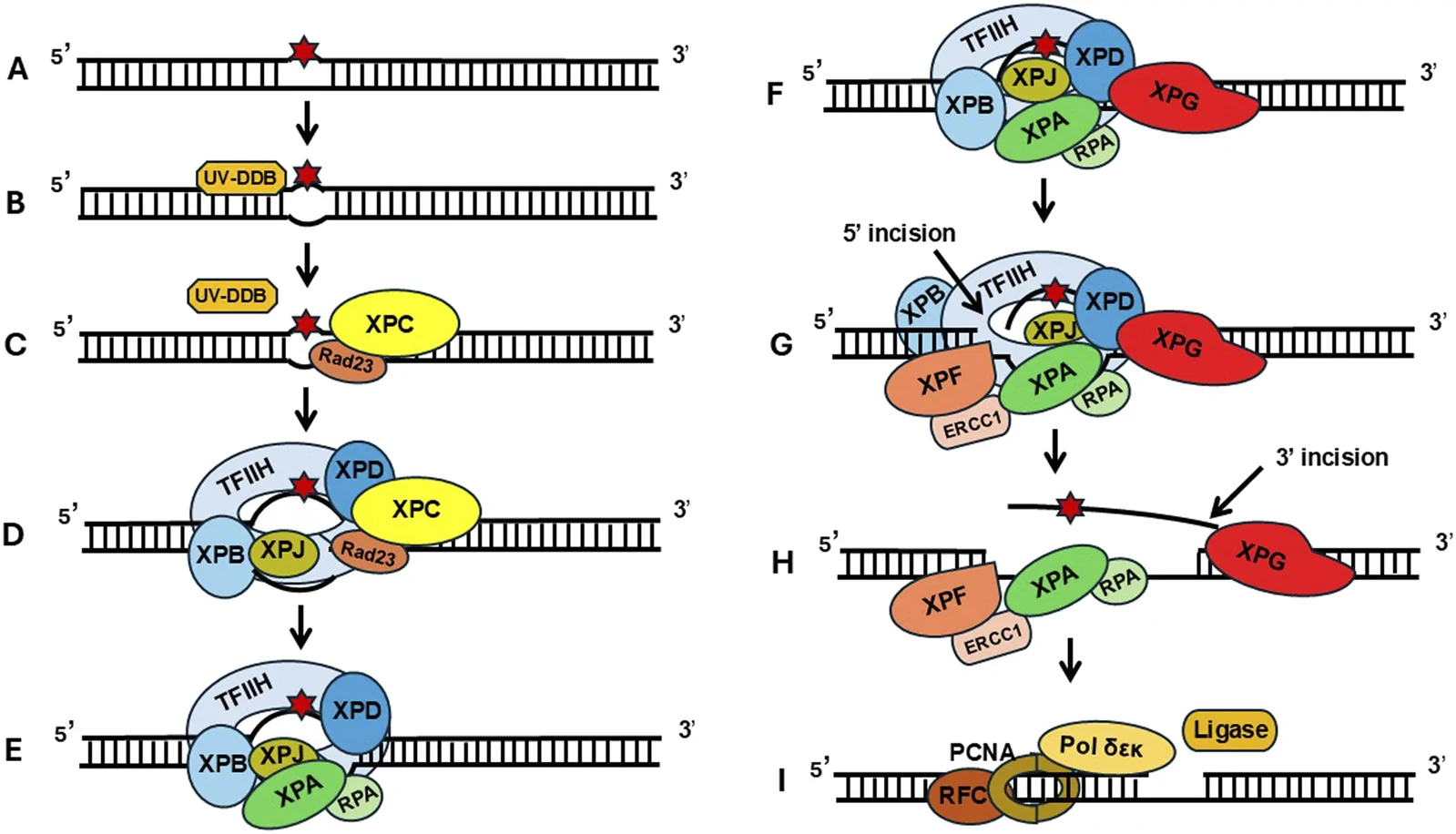
GG-NER of UV-irradiation induced DNA damage in normal human cells. **(A)** Cyclobutane pyrimidine dimers or (6-4)PPs are induced in DNA. **(B)** DDB2 (XPE) recognizes and binds to the DNA at the site of damage. **(C)** XPC in a complex with RAD23B is recruited to the site of damage. DDB2 undergoes ubiquitination and degradation and dissociates from the DNA. **(D)** The XPC complex recruits TRIIH, with subunits XPB, XPD and XPJ to the area of DNA damage. TFIIH opens the DNA helix around the lesion. **(E)** XPA binds to the damaged DNA and to TFIIH and stimulates the helicase activity of XPD. XPA together with RPA stabilizes the pre-incision complex and aids in the accurate assembly and positioning of additional proteins needed in the repair process. XPA helps in the translocation of XPB/TFIIH along the DNA in a 5′ to 3′ direction and in coordination of the verification of the DNA damage. **(F)** XPG is recruited to the complex and is needed for recruitment of XPF-ERCC1. **(G)** XPF creates the first incision 5′ to the lesion. This is followed by the second incision 3′ to the lesion by XPG. **(H)** This leads to excision of a 22-32 nucleotide containing the damage. **(I)** The resulting gap is filled by DNA synthesis by replication proteins RFC, PCNA, a DNA polymerase (δ,ε, or κ) and sealed by a DNA ligase.

In GG-NER of UV-induced CPDs and (6-4)PPs in normal human cells, the lesion is first recognized by UV-DDB (XP-E), a UV-damage DNA-binding protein complex comprising DDB1, DDB2, CUL4A and RBX1, which has high affinity for these lesions [34, 42, 44–48]. UV-DDB binds to the DNA at the site of damage introducing an opening into the DNA duplex and stimulating recruitment of XPC (Figure 1B) [34, 44–47]. After damage recognition, DDB2 undergoes ubiquitination by a E3 ubiquitin ligase activity present in UV-DDB and this leads to its degradation and dissociation from the DNA [46, 48–50]. This, in turn, facilitates recruitment of XPC to the site of damage (Figure 1C). XPC is part of a heterotrimeric complex with RAD23B and Centrin-2 (CENT2), which help stabilize XPC and promote its binding to the site of damage [42, 51, 52]. XPC’s primary role is in recognition of the DNA lesion. XPC-RAD2B-CENT2 recruits TFIIH to the area of the DNA lesion (Figure 1D) [48, 53]. TFIIH consists of 10 subunits which include a core complex (p62, p52, p44, p43 and p8), the CAK complex (MAT1, CDK7, and cyclin+1) and XPB and XPD [54, 55]. XPC interacts through its C-terminal domain with XPB, a subunit of TFIIH, and through its N-terminal with the p62 subunit of TFIIH [48, 53, 56–58]. The p52 subunit of TFIIH (XP-J) is important for recruitment of TFIIH to the damaged DNA through its interaction with XPC and XPA (Figure 1D) [31, 57, 58]. XPB, in the TFIIH complex, binds 5′ to the lesion where it initiates opening of the DNA bubble via its translocation activity and tracks along DNA in a 5′ to 3′ direction expanding the bubble and unwinding the DNA around the lesion during NER [48, 56–60]. XPD, another subunit of TFIIH, is a 5′ to 3′ helicase which tracks along the DNA scanning it in a 5′ to 3′ direction and is responsible for lesion verification, DNA unwinding and extension of the repair bubble [48, 56–61]. The helicase activity and damage verification by XPD is stimulated by the XPA protein, which binds to the damaged DNA and to TFIIH on the 5′ side of the lesion where it forms an arch over the DNA that bridges XPB and XPD (Figure 1E) [30, 31, 42, 56–58]. The interaction of XPA with TFIIH helps in the translocation of TFIIH and XPD along the DNA in a 5′ to 3′ direction and in coordination of the verification of DNA damage that occurs in NER [30, 31, 48, 56–58]. XPA, associates with RPA32 and RPA70 to form a complex which stabilizes the pre-incision complex and provides a scaffold which aids in the accurate assembly and positioning of additional proteins needed in the repair process (Figure 1E) [18, 42, 46, 56, 62–66]. These processes need to be completed before incision can take place [54–56, 65–67]. Two endonucleases, XPF-ERCC1 and XPG, then create incisions on either side of the lesion. XPG, which has tight interactions with TFIIH, is recruited first to the complex and needs to be present for the recruitment of XPF-ERCC1 to the site of damage (Figure 1F) [54, 56, 66, 68–71]. This recruitment occurs through the direct interaction of XPA with ERCC1 and results in the production of the first incision 5′ to the lesion by XPF (Figure 1G) [48, 54, 56, 65, 68–71]. This is followed by the second incision 3′ to the lesion produced by XPG, which leads to excision of the damage (Figure 1H) [48, 56, 63]. A 22-32 nucleotide is released and the resulting DNA gap is filled by DNA synthesis which involves the activity of replication proteins RFC, PCNA and a DNA polymerase, which may vary: DNA polymerase δ (non-replicating cells), ε (mainly in replicating cells), or κ (non-replicating cells) (Figure 1I) [42, 48, 52, 56, 67, 72]. The gap is sealed by either DNA ligase I or ligase III.

### Defects in XP variant cells in DNA replication past sites of damage

In one group of XP patients, the XP variants (XP-V), the cells are not deficient in NER (Table 1). They have a deficiency in ability to bypass DNA lesions, such as cyclobutene pyrimidine dimers during DNA replication, and are defective in translesion DNA synthesis [5, 16, 17, 73–76]. XP-V cells have mutations in the *POLH* gene, which encodes a DNA polymerase, pol η, that is associated with DNA replication during S phase [5, 15, 17, 75]. This polymerase has a relatively low stringency which facilitates translesion synthesis past different types of DNA damage [5, 15, 17, 75]. Pol η is the major and most efficient way for cells to bypass UV-induced cyclobutene pyrimidine dimers [18, 76]. In the absence of this protein, a less efficient and more error-prone polymerase may carry out DNA synthesis past the lesion but this leads to increased mutations after UV radiation [5, 17, 18].

Approximately 23% of patients with XP are XP variants [5, 16]. XP-V patients have a typical XP phenotype and generally have milder symptoms and do not have major neurological abnormalities [5, 15–17], However, XPV cells are extremely hypermutable by UV-irradiation and this could account for the skin cancer susceptibility of the XP-V patients and to some of the clinically severe cases where patients have developed a number of tumors [5, 15–17]. Thus, this group of XP patients is sensitive to UV induced damage in the skin, however, the clinical characteristics of XP-V patients, though similar to those of the XP-A to XP-J patients, are not due to a DNA repair defect in NER, but to a defect in ability to accurately replicate DNA past a site of damage [5, 15–17, 73, 75]. This emphasizes the genetic diversity that exists in this group of patients.

## The importance of XPA in NER of DNA damage

XPA plays a critical role in NER. Its importance in the repair process is seen in patients in the XP-A complementation group who are among the most severely affected and have some of the lowest levels of repair of UV-induced DNA lesions [3, 4, 6, 8]. Levels of UDS in cells form these patients are usually 2%–5% of normal, though there is variability (Table 1) [3, 4]. Deficiencies in the XPA protein, due to mutations in the *XPA* gene, are responsible for the clinical heterogeneity observed in these patients. XPA is the smallest of the XP proteins, 31 kDa, and it interacts with DNA through a minimal DNA binding domain which is present in the central globular core and extends into the C-terminal domain [26, 27, 29, 77, 78]. It was originally thought to be the damage recognition protein, but this role is now attributed to XPC/DDB (DNA damage binding protein) [79]. XPA contains sites for interaction with a number of NER proteins. It interacts with TFIIH and helps clamp the TFIIH helicases, XPB and XPD, in the proper position for their catalytic function and prevents XPB from detaching from the DNA during NER Figure 1F) [18, 28, 30, 31, 42, 54, 56, 58, 80]. The C-terminal domain of XPA binds to three subunits of TFIIH (p8, p52, and XPB) [28, 56]. The p52 subunit has now been identified as XPJ [11, 12]. The N-terminal domain interacts with the XPD subunit of TFIIH [28, 56]. XPA interacts with RPA through two binding motifs. The primary site is in the N-terminal domain of XPA which interacts with RPA 32, which is important in the recruitment and localization of XPA at the site of damage [28, 52, 56, 58, 80]. The secondary site interaction involves the binding of RPA 70A and RPA 70B to the Zn binding subdomain of the DNA binding central domain [28, 52, 56, 58, 80]. This binding helps position XPA and RPA at the site of damage and guide the positioning of other NER proteins at this site [28, 52, 56, 58, 80]. XPA’s direct interaction, in its N-terminal region, with ERCC1 enables the recruitment of XPF-ERCC1 and, along with XPG, which is associated with TFIIH, this allows the dual incisions to occur at the damage site [29, 42, 56, 78, 81]. The multitude of interactions of XPA with these and other repair proteins makes it an important scaffold bringing about distinct protein-protein interactions between key components of NER and making it a significant coordinator of NER dynamics [27, 30, 34, 56].

XPA has, in addition, been proposed to have another important role in NER. Lambert et al have proposed that XPA enables endonucleases involved in NER (XPF and XPG) to localize to sites of damage on DNA using a processive mechanism of action [25, 32]. They have shown that this function of XPA is particularly important in repair of damaged nucleosomal DNA [25, 32]. Additionally, more recent studies by Kovic et al and Kappenberger et al. have indicated that XPA from the eukaryotic fungus, *Chaetomium thermophilum,* is also involved in increasing the processivity of the XPB/TFIIH translocase, as it translocates along UV-damaged DNA aiding in its unwinding during NER [30, 31]. XPA could thus have an important role in mechanistically enhancing progesssivity of several proteins involved in NER. This will be discussed in more detail below.

## Influence of XPA on mode of action of DNA endonucleases in localizing sites of damage

### Action of XPA as a processivity factor

Determination of the mechanisms DNA repair proteins utilize to locate and interact with their target sites is critical for elucidating how DNA repair processes are regulated. There are two distinct ways that proteins can locate target sites on DNA: (1) a processive mechanism of action in which a protein randomly binds to a nonspecific site on DNA and then locates its recognition sites by a one-dimensional facilitated-scanning process where it slides or hops along the DNA by a one-dimensional diffusion mechanism to locate target sites before dissociating from the DNA, or (2) a distributive mechanism of action in which a protein has no affinity for non-target DNA and uses a random, three-dimensional search process to locate target sites where there is diminution of protein-nontarget DNA interactions [82–84]. Whether a protein acts by a processive or distributive mechanism has been shown to be of marked biological significance. A number of proteins involved in DNA repair act by a processive mechanism of action. A processive mechanism of action of a protein can significantly increase the rate and efficiency of location of target sites within large domains of non-target DNA [82, 83, 85]. The processivity of the protein can be mediated by the protein itself as occurs for apurinic/apyrimidinic endonucleases, DNA glycosylases, T4 endonuclease V, UvrABC nuclease, and *Micrococcus luteus* UV endonuclease [83, 85–92]. Replicative DNA polymerases (i.e.,pol delta and pol epsilon), also have high processivity and high fidelity whereas a translesion DNA polymerase, such as pol η, has a highly distributive mode of synthesis and a relatively low stringency which facilitates translesion synthesis past different types of DNA damage [15, 75, 93]. The processivity of a protein can also be mediated by a separate accessory protein as is the case for *E. coli* DNA polymerase III [94] and bacteriophage T4 DNA polymerase [95, 96].

XPA is a protein which mediates a processive mechanism of action for several proteins involved in NER. Lambert et al. have shown that XPA enables endonucleases from several normal human cell lines involved in NER (XPF and XPG) to locate sites of damage on DNA using a processive mechanism of action [25, 32]. More recently, studies by Kovic et al and Kappenberger et al., using the eukaryotic fungus, *Chaetomium thermophilim*, as a model system, indicate that XPA increases the processivity of the TFIIH translocase, XPB [30, 31]. This will be discussed in more detail below. The proposed ability of XPA to aid in the processive translocation of several proteins involved in NER may enhance the efficiency of the repair process.

### Differences between normal and XP-A cells in mechanism of localization of sites of damage by DNA endonucleases

**FIGURE 2:**
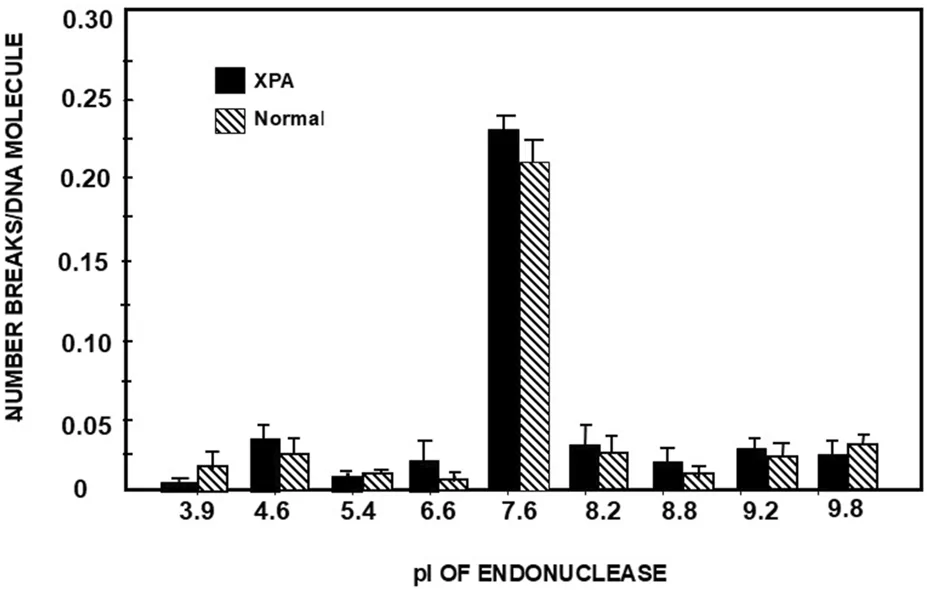
Activity of chromatin-associated DNA endonuclease complexes from XPA and normal human cells on UVC light irradiated DNA. Endonuclease activity was assessed on DNA irradiated with 45 J/m^2^ UVC light. These values have had subtracted from them the enzyme activity on undamaged DNA (0.05 ± 0.01 breaks). Vertical lines represent ±SEM of six experiments from two different normal and two different XPA cell lines (Modified from Feng et al. [32] with permission from Oxford University Press).

An endonuclease complex has been isolated from the nuclei of normal human lymphoblastoid cells by Lambert et al. which has an isoelectric point (pI) of 7.6 [32, 97–99]. It recognizes and incises DNA containing predominately cyclobutene pyrimidine dimers produced by UVC-irradiation [32, 97–99]. This complex contains proteins involved in the NER pathway and includes XPA, RPA, TFIIH (XPB, XPD, p34, p44, and p62), hHR23B, XPF, ERCC1, XPG and PCNA [83, 84]. Using an isolated *in vitro* system and UVC-irradiated plasmid DNA, they have shown that the endonucleases in this complex from two different normal human cell lines, which are presumed to be XPF and XPG, incise DNA using a processive mechanism of action [32, 99]. Of considerable interest, the endonuclease complex, pI 7.6, from two different XP-A cell lines, was found to incise the damaged DNA using a distributive mechanism of action [32, 99]. Both the normal and the XP-A endonuclease complexes, over a time course, produced a similar number of incisions on UVC-irradiated DNA (Figure 2) [32, 99]. Both the normal and XP-A endonucleases had over three times the activity on damaged DNA compared to undamaged DNA; thus, there was no deficiency in the ability of the XP-A endonucleases to incise the UVC-irradiated naked DNA (Figure 2) [32, 99]. This would suggest that whether the endonucleases act by a processive or distributive mechanism of action does not affect their ability to incise damaged naked DNA.

The mechanisms utilized by the endonucleases to locate sites of damage was determined using two different methods. In the first method, a circular, supercoiled plasmid DNA containing the entire SV40 and pBR322 genomes was used as substrate and nicking assays were carried out [32, 98]. The basis of this assay is that if an endonuclease is acting by a processive mechanism of action, it will bind nonspecifically to DNA and scan the DNA making incisions at sites of damage as they are encountered. The supercoiled DNA will decrease, nicked circular DNA will increase and then linear DNA will be produced as nicks occur in proximity on both DNA strands [85]. This will increase linearly over time and this was observed with the normal endonuclease complex [32]. If an endonuclease is using a distributive mechanism of action, a three-dimensional search will occur in which random incisions are made at the site of damage. The supercoiled DNA will decrease with time but accumulation of the linear DNA will lag until enough random breaks are made in the DNA to produce a linear DNA molecule. This was observed with the XP-A endonuclease complex [32, 99].

**FIGURE 3:**
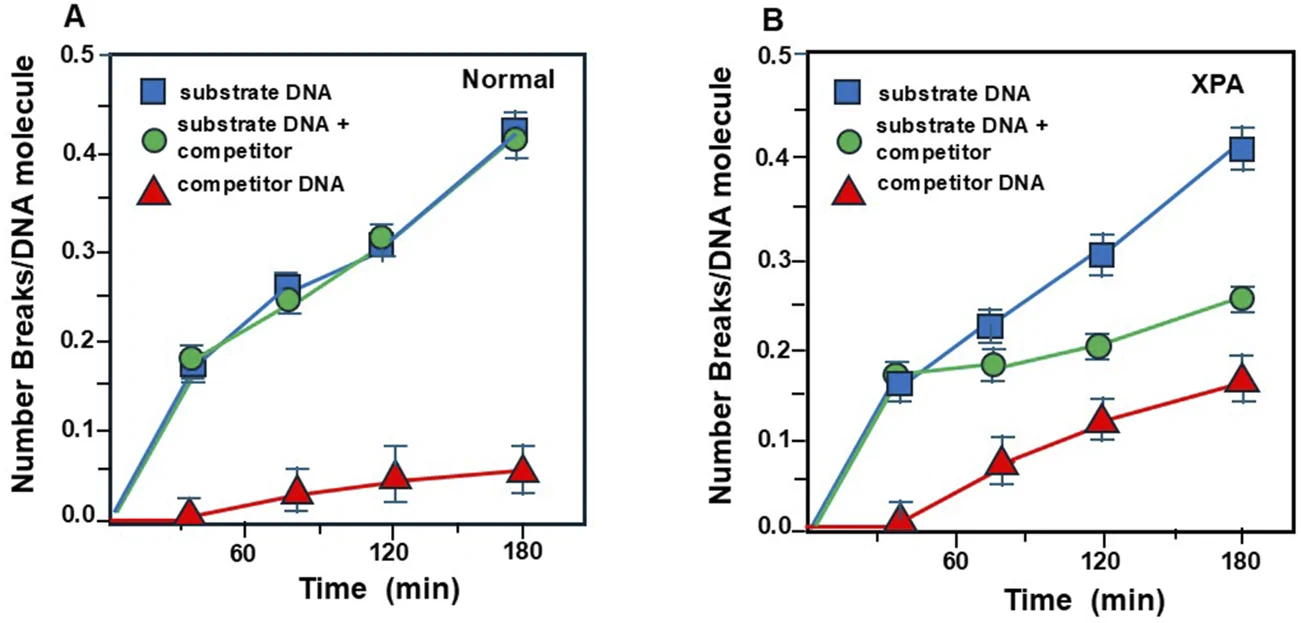
Analysis of normal and XPA endonuclease incision of UVC-irradiated substrate DNA in the presence of a competitor DNA. The normal **(A)** and XPA **(B)** endonucleases complexes were incubated with UVC irradiated plasmid DNA (pWT830/pBR322) for 40 min. A UVC irradiated competitor DNA (pGM-3zf) was then added to the reaction and incubation continued for the indicated times. The number of breaks per DNA molecule that each endonuclease produced on the substrate and competitor DNAs was calculated. Endonuclease activity on: 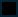 substrate DNA; 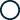 substrate DNA in the presence of competitor DNA; 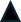 competitor DNA, Vertical lines represent ±SEM for four experiments using two different normal and two different XP-A cell lines (Modified from Feng et al. [32] with permission from Oxford University Press).

The basis of the second method for determination of target site location by the endonucleases was use of a competitor DNA in the assay [32]. If an endonuclease is acting in a processive manner, it will scan the DNA, incising at multiple sites before it dissociates from the DNA and then associates with a different DNA molecule. When a competitor DNA is added to the assay, after the endonuclease has had time to initially bind to the substrate DNA, the endonuclease will have little activity on the competitor while it is still active on the substrate DNA. Addition of the competitor will, therefore, have little effect on the activity of the endonuclease on the substrate DNA [32]. This was found to be the case with the endonuclease complex from normal cells (Figure 3A) [32]. However, if the endonuclease is acting in a distributive manner, it will randomly search and incise both the substrate DNA and the competitor DNA at sites of damage. The activity of the endonuclease on the substrate DNA will rapidly decrease when the competitor is added since the endonuclease is also producing incisions on the competitor DNA. This was found to occur when the XP-A endonuclease complex was examined (Figure 3B) [32]. Use of both of these methods of assay indicates that the normal endonucleases examined locate sites of damage by a processive mechanism of action and the XP-A endonucleases in the cell lines examined do so by a distributive mechanism of action [32, 99]. Irrespective of their mechanism of localization of sites of DNA damage, both the normal and XP-A endonucleases had similar levels of activity on the UVC-irradiated naked DNA (Figures 3A,B) [32, 99]. There is disagreement between these findings and other reported studies [100, 101]. However, several important factors need to be taken into account when examining these studies, which will be discussed in a later section.

**FIGURE 4:**
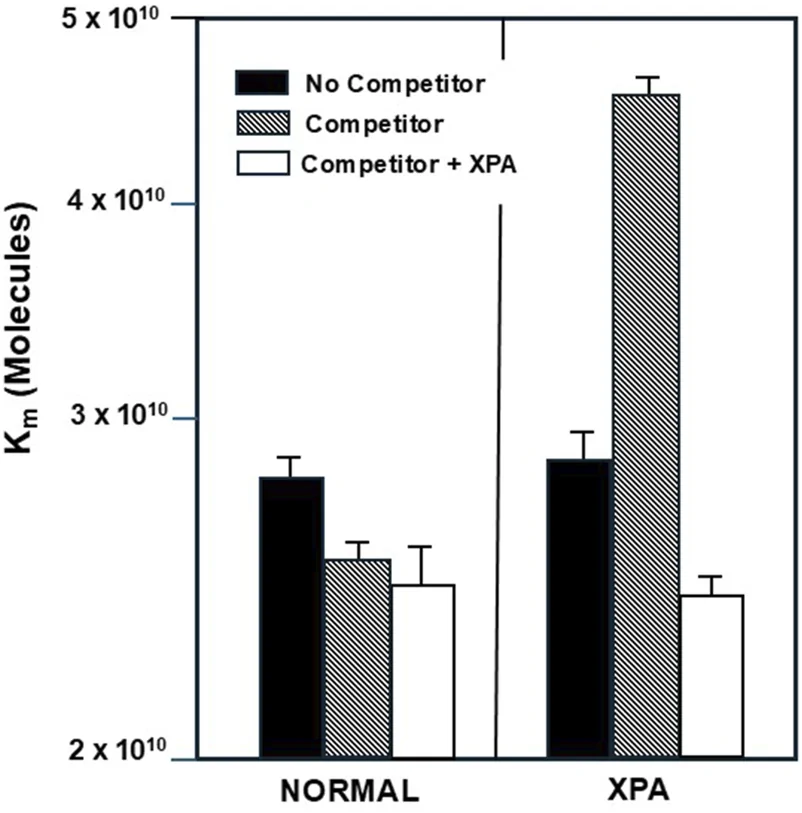
Kinetic analysis of the activity of the normal and XPA endonucleases on UVC irradiated DNA and the influence of a competitor DNA and a competitor DNA in the presence of a recombinant XPA protein on this activity. K_m_s of the endonucleases in the normal and XPA complexes on UCV- irradiated DNA was determined, either in the absence of UVC-irradiated competitor DNA, with addition of the competitor DNA, or with both the competitor DNA and recombinant XPA protein. Reaction conditions were as in Figure 3. Vertical lines represent ±SEM for 3-4 experiments (Modified from Lambert and Yang [25] with permission from Academic Press).

Kinetic analysis was also carried out on the activities of the endonucleases from the normal and XP-A complexes on UVC-irradiated DNA [25]. These studies have shown that the K_m_s of the normal and XP-A endonucleases on UVC-irradiated DNA, in the absence of a competitor DNA, were similar, indicating that they have similar affinity for or rate of association with the damaged DNA (Figure 4) [25]. This confirmed the finding that there is no deficiency in the ability of the endonucleases from these XP-A cells to incise damaged naked DNA [25]. However, in the presence of a competitor DNA, the K_m_s of the XP-A endonucleases, but not the normal endonucleases, increased approximately 60% on UVC-irradiated DNA compared to the K_m_s of these endonucleases on UVC-irradiated DNA when no competitor was present (Figure 4). [25, 32]. This indicates that the XP-A endonucleases but not the normal endonucleases have a reduced rate of association with or affinity for UVC-damaged DNA when a competitor DNA is present. This is consistent with these XP-A endonucleases utilizing a distributive mechanism of action and the normal endonucleases utilizing a processive mechanism of action for localization of sites of damage on the DNA [25, 32]. It provides further evidence that the normal and XP-A endonucleases examined in these studies localize sites of damage by different mechanisms.

### The XPA protein can correct the inability of endonucleases from XP-A cells to localize damage sites by a processive mechanism of action

**FIGURE 5:**
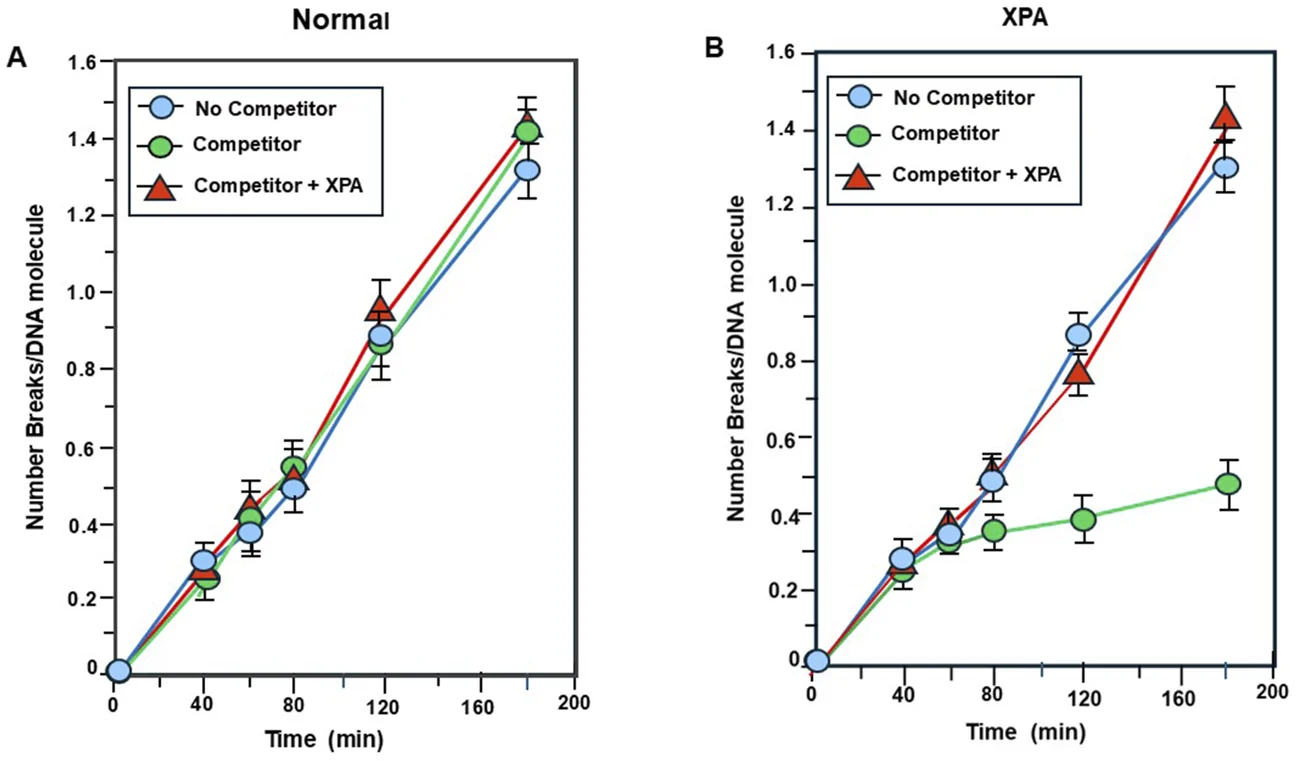
Influence of recombinant XPA on the activity of endonucleases in the normal and XPA complexes on UVC-irradiated DNA in the presence of a similarly damaged competitor DNA. **(A)** The normal endonuclease complex either with or without XPA was incubated with UVC-irradiated plasmid DNA for 40 min. **(B)** The XPA endonuclease complex either with or without XPA was incubated with UVC-irradiated plasmid DNA for 40 min. To both sets of samples, the UVC-irradiated competitor DNA was then added and the incubation continued for the indicated times. The number of endonuclease mediated breaks per DNA molecule was calculated. Vertical lines represent ±SEM for 3 to 4 experiments (Modified from Lambert and Yang [25] with permission of Academic Press).

In order to further demonstrate that XPA is important in the processive mechanism of action of the endonucleases involved in NER, Lambert et al. carried out studies to determine whether a recombinant XPA protein could switch the endonucleases in the XP-A cells utilized in these studies from a distributive to a processive mechanism of action [25]. For this analysis, a recombinant XPA protein was produced by expression of the normal human *XPA* cDNA in *E. coli* followed by its extraction and purification [25]. A plasmid DNA containing the entire SV40 and pBR322 genomes, was used as the substrate and was irradiated with UVC light so as to produce approximately 25 pyrimidine dimers per DNA molecule [25, 32]. Analysis of endonuclease activity was carried out using assays in which a competitor DNA was added to the reaction [25]. The results showed that, when the purified recombinant XPA protein was added to the reaction, the endonucleases in the XP-A complex switched to a processive mechanism of action and were able to scan and produce incisions mainly on the substrate DNA even in the presence of a competitor DNA (Figure 5B) [25]. This is an indication that the XPA protein is acting as a processivity factor enabling the XP-A endonucleases to utilize a processive mechanism of action. Addition of the purified recombinant XPA to the reactions, which contained the normal endonuclease complex, had little effect on the activity of the normal endonucleases on the damaged substrates. These endonucleases continued to act in a processive manner and produced incisions on the substrate DNA in the presence of the competitor DNA (Figure 5A).

Kinetic analysis of the influence of the recombinant XPA protein on the activity of the endonucleases in the XP-A complex was also carried out [25]. As previously shown, the K_m_ of the normal and XP-A endonucleases were similar on UVC-irradiated DNA in the absence of the competitor (Figure 4). When the recombinant XPA protein was added to the reaction along with the competitor DNA, the Km of the normal endonucleases on the damaged substrate was similar to the Km of the endonucleases when only the competitor DNA was present (Figure 4). However, for the XPA endonucleases, when the recombinant XPA was added to the reaction along with the competitor DNA, the K_m_ of the XP-A endonucleases was greatly reduced, compared to the Km when only a competitor DNA was present, and was similar to that of the normal endonucleases, when both the competitor DNA and recombinant XPA were present (Figure 4). This indicates that the recombinant XPA protein was able to restore the deficiency in the ability of the endonucleases present in the XP-A complex to locate sites of damage by a processive mechanism of action. Since the endonuclease complex. pI 7.6, contains the endonucleases XPF and XPG, it is proposed that these are the endonucleases creating the incisions in the work described above. Based on these studies, it has been proposed that another important role of the XPA protein in NER is that of a processivity factor enabling the endonucleases involved in NER, presumably XPF and XPG, to incise DNA using a processive mechanism of action [25, 99]. Whether this deficiency in the XPA protein is present in a larger number of XPA cells lines will need to be determined and may depend upon specific mutations in the XPA gene, particularly in the DNA binding domain which is important in strengthening binding of XPA to DNA during the repair process [26, 27].

## XPA increases the processivity of the XPB core TFIIH complex as its translocates along damaged DNA

Studies also suggest that XPA increases the processivity of the translocase activity of XPB/coreTFIIH on damaged DNA [30, 31]. The eukaryotic fungus, *Chaetomium thermophilum,* was used as a model system for examining this since its proteins have high structural homology with human proteins [30, 31]. These studies indicated that XPA increases the processivity of the XPB core TFIIH complex as it translocates along DNA in a 5′ to 3′ direction enhancing the unwinding of the DNA repair bubble during NER [30, 31]. XPA crosslinks to XPB and it has been proposed that XPA facilitates the lesion scanning and the helicase activity of XPB on the damaged DNA [30, 31]. Another study showed that purified recombinant human XPA protein stimulated the TFIIH helicases, XPB and XPD, and DNA unwinding as they translocated along damaged DNA [30]. These studies suggested that XPA may clamp XPB/core TFIIH onto the DNA during lesion scanning enhancing the ability of XPB to processively translocate along DNA in the unwinding process [30, 31]. Since XPG is associated with the XPB/TFIIH complex during the translocation process, it has been suggested that this may increase the availability of XPG for the incision steps in the repair process [30, 31]. Also, since XPA chaperons TFIIH-DNA interactions and anchors the NER machinery to the repair bubble, it has also been suggested that XPA is ideally positioned to recruit XPF-ERCC1 and thus aid in the assembly of the two endonucleases, XPF-ERCC1 and XPG, at the site of the lesion so they can incise the DNA [30, 31].

There are now two studies which suggest that XPA may play an important role in enhancing the processivity of several proteins involved in the NER process [25, 30–32]. In one case, XPA is proposed to enhance the ability of endonucleases involved in the incision process (XPF and XPG) to locate sites of damage by a processive mechanism of action [25, 32, 33, 99]. In the second case, XPA is proposed to enhance the processivity of XPB/core TFIIH as it translocates along the damaged DNA unwinding it and enabling further steps in the repair process [30, 31]. It could further be proposed that the enhanced processivity of these repair proteins, should increase their ability to associate with and scan the damaged DNA and facilitate the speed and accuracy of the repair process. Whether the proposed role of XPA in the processive movement of XPB/core TRIIH complex along damaged DNA is related to XPA’s proposed role in the processive mechanism of action of XPG and XPF in locating sites of damage should be explored further, particularly since one process usually follows the other in the NER repair pathway.

It would also be of considerable interest to determine whether there is a deficiency in the ability of XPA to enhance the processive translocation of XPB/core TFIIH along UV-damaged DNA in XP-A cells. If a defect were found in ability of XPB to processively traverse along the damaged DNA, this would give an indication of the importance of XPA as a processivity factor for several proteins essential for in NER.

## Role of XPA as a processivity factor is critical for production of incisions on damaged nucleosomal DNA

Within the cell, genomic DNA is packaged into chromatin and this has been shown to have an impact on the ability of proteins involved in processing of DNA to interact with their target sites and includes proteins involved in DNA repair, DNA replication and DNA transcription. Chromatin structure plays a significant role in the DNA repair process where it can determine the distribution of sites of damage as well as their accessibility to repair proteins [99, 102–109]. In chromatin, the DNA is wrapped around histone octamers (H2A, H2B,H3, H4) to form nucleosome core particles, which are connected by linker DNA [110–113]. Histone H1 binds to the nucleosome at the entry and exit sites of the linker DNA [110–113]. Nucleosome structure can thus have a significant effect on DNA repair and can modulate NER activity [32, 33, 98, 99, 102–109].

### Normal and XP-A endonuclease activity on damaged nucleosomal DNA

Studies have been carried out to examine whether nucleosome structure has an effect on the activity of the endonucleases from normal and XP-A cells on damaged nucleosomal DNA. An *in vitro* system was developed by Lambert et al. that consisted of nucleosomes reconstituted with plasmid DNA containing both the SV40 and pBR322 genomes and either core (H2A, H2B, H3, and H4) or total (core plus H1) histones purified from normal or XP-A lymphoblastoid cells [32, 98, 114]. This plasmid was used so that a comparison of the reconstituted nucleosomal substrate could be made with the SV40 minichromosome. This system allowed regulating the number of nucleosomes reconstituted per DNA molecule and provided a means to quantitate the number of endonuclease-mediated incisions on UVC-irradiated nucleosomal DNA with the number of nucleosomes per DNA molecule [32, 98, 114]. Examination of the reconstituted nucleosomal substrate after UVC-irradiation showed that the number of pyrimidine dimers in core and total nucleosomal DNA was 4.7 and 4.6, respectively, which were 10% and 12% less than were present in damaged naked DNA [32]. The number of nucleosomes per DNA molecule was 23 for either core or total nucleosomal DNA [32]. This number could be regulated by changing the ratio of histones-DNA used [32].

**FIGURE 6:**
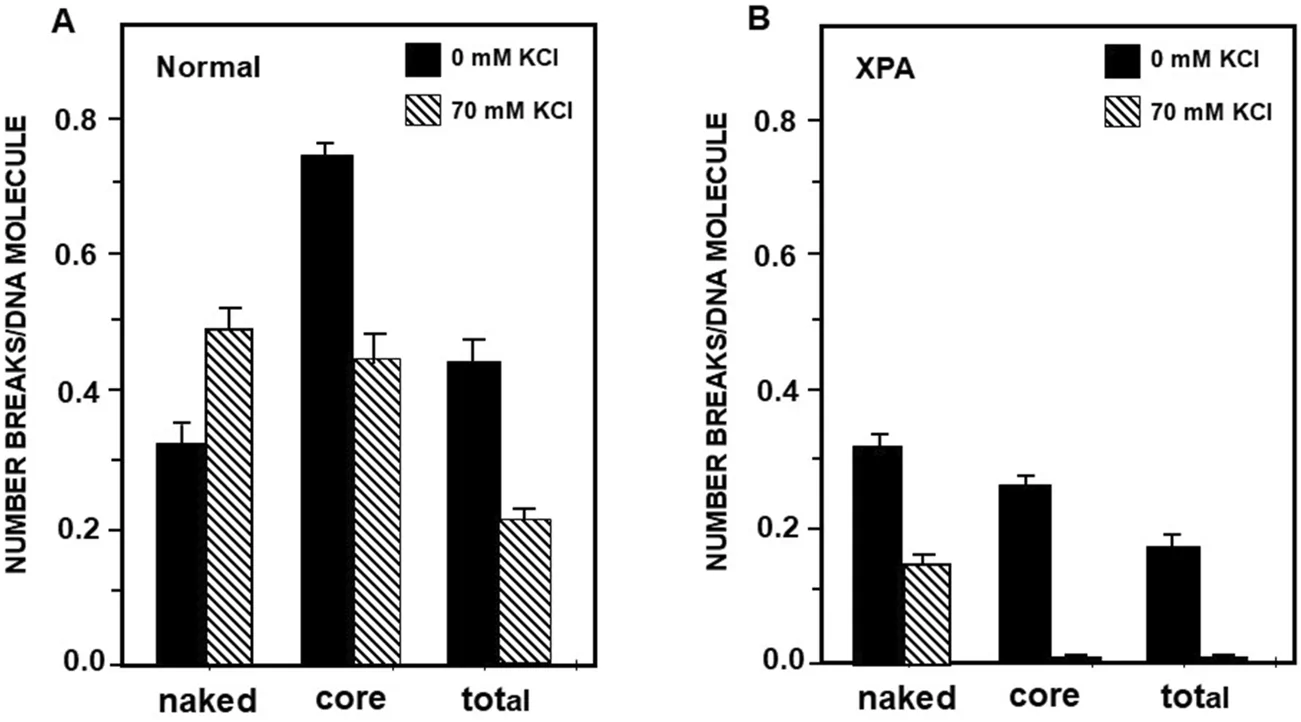
Activity of the DNA endonucleases form normal and XP-A cells on reconstituted nucleosomal DNA and the influence of KCl concentration on this activity. The normal **(A)** and the XPA **(B)** endonuclease complexes were incubated for 180 min with UVC-irradiated naked DNA or DNA reconstituted at a 1:1 ratio with core or total histones. The reaction solution had a KCl concentration of 0 mM (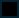) or 70 mM (shaded square). The number of breaks per DNA molecule was determined. Vertical lines represent ±SEM for five experiments. Two different normal and two different XP-A cell lines were used (Modified from Feng et al. [32] with permission from Oxford University Press).

The results of these studies demonstrated that there was a difference in the activity of the endonucleases from normal cells on damaged nucleosomal DNA compared to the activity of these endonucleases on damaged naked DNA. The endonucleases from normal cells had 2.3-fold increased activity on damaged core nucleosomal DNA compared to damaged naked DNA (Figure 6A) [32]. This increase was reduced to 1.4-fold when histone H1 was added to the system (Figure 6A) [32]. The reduction in the increased endonuclease activity on damaged nucleosomal DNA when histone H1 was add to the system agrees with the role of histone H1 in the condensation of chromatin making it less accessible to endonucleolytic attack [115, 116]. The increase in endonuclease activity on core nucleosomal DNA occurred even though the number of pyrimidine dimers on the nucleosomal DNA was less than on the naked DNA. This increase correlated with the number of nucleosomes present on the DNA and increased linearly as the number of core or total nucleosomes was increased [32]. Similar results of increased endonuclease activity on damaged nucleosomal DNA were obtained when a DNA endonuclease complex, pI 4.6, from normal human cells, which has specificity for DNA interstrand cross-links, was examined for activity on 8-methoxypsoralen (8-MOP) plus UVA light damaged core and total nucleosomal DNA [98]. Though different damage recognition proteins are involved in repair of both types of damage, endonucleolytic incision is involved in DNA ICL repair just as is in NER [99]. Thus, this increase in endonucleolytic activity in normal cells on damaged nucleosomal DNA is not due to a specific type of DNA damage.

The endonucleases from the endonuclease complex, pI 7.6, in XP-A cells had slightly decreased incision activity on damaged core nucleosomal DNA compared to its activity on damaged naked DNA and approximately 50% decreased activity on damaged nucleosomal DNA when histone H1 was added (Figure 6B) [32]. The activity of these endonucleases was also depended on the number of nucleosomes on the DNA. As the number of nucleosomes increased, the XP-A endonuclease activity decreased unlike the increase in endonuclease activity that was observed in the normal endonuclease complex [32]. Thus, unlike the normal endonuclease complex, the endonucleases in the XP-A complex were defective in ability to incise UVC-damaged nucleosomal DNA [32].

### A processive versus a distributive mechanism of localization of sites of damage by normal and XP-A endonucleases on nucleosomal DNA

**FIGURE 7:**
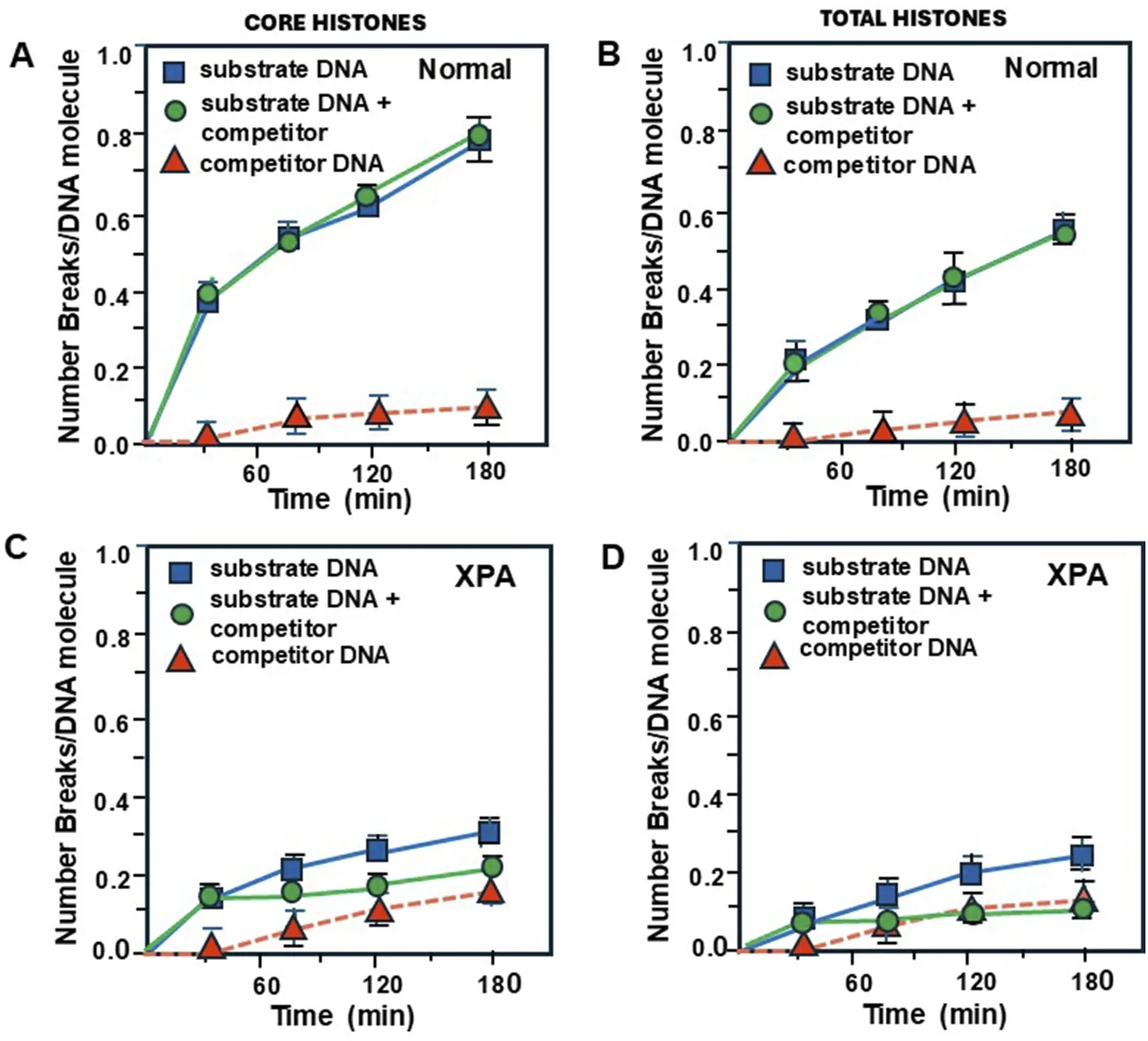
Analysis of the activity of the normal and XPA DNA endonucleases on UVC-irradiated nucleosomal DNA in the presence of a competitor DNA. The normal complex was incubated for 40 min. with UVC-irradiated plasmid DNA reconstituted with: **(A)** core histones or **(B)** total histones, at a histone/DNA ratio of 1. The XPA complex was similarly incubated with UVC-irradiated plasmid reconstituted with: **(C)** core histones or **(D)** total histones. UVC-irradiated competitor DNA was then added and the incubation continued for the indicated times. The number of breaks/DNA molecule was determined as in Figure 3. Endonuclease activity on: 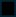 substrate DNA; 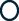 substrate DNA in the presence of competitor DNA; 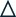 competitor DNA, Vertical lines represent ±SEM for four experiments using two different normal and two different XP-A cell lines (Modified from Feng et al. [32] with permission from Oxford University Press).

Studies were undertaken to determine whether both the normal and XP-A endonucleases continued to use the same mechanism of action (i.e., processive and distributive, respectively) on the UVC damaged nucleosomal DNA as they did on damaged naked DNA [32]. The number of incisions produced by the normal endonucleases on either UVC-irradiated core or total nucleosomal DNA increased compared to their activity on UVC damaged non-nucleosomal DNA, but this activity did not change when a competitor was added, indicative of a processive mechanism of endonuclease action (Figures 7A,B) [32]. In contrast, the number of incisions produced by the XP-A endonucleases on UVC-irradiated nucleosomal DNA was significantly reduced compared to their activity on damaged non-nucleosomal DNA when a competitor DNA was present [32]. The number of incisions created on UVC-irradiated core or total nucleosomal DNA was only 60% and 50%, respectively, of the number produced in the absence of a competitor DNA (Figures 7C,D) [32]. The level of cleavage of competitor DNA was approximately 2.5 times greater than the levels of cleavage of competitor DNA by the normal endonucleases [32]. This indicates that the XP-A endonucleases are locating sites of damage on nucleosomal DNA in a distributive manner just as they do on UVC damaged non-nucleosomal DNA [32, 99]. Whether normal or XP-A histones were used in the reconstituted nucleosomal system did not make a difference [32, 99]. These studies suggest that the ability of these endonucleases to incise damaged nucleosomal DNA is related to the mechanism they use to localize sites of damage (i.e., whether it is by a processive or distributive mechanism of action) [32]. Endonucleases which locate their sites of damage by a processive mechanism of action, such as the normal endonucleases, are able to incise damaged nucleosomal DNA, whereas, endonucleases which utilize a distributive mechanism of action, such as the XP-A endonucleases are deficient in this ability.

The results of the studies of Lambert et al. showing that XP-A endonucleases can incise damaged naked DNA but not damaged nucleosomal DNA are in agreement with the findings of Mortelmans et al. and Kano and Fujiwara which indicated that XP-A cells are defective in a factor that renders DNA in UV-irradiated chromatin accessible to incision by cellular enzymes [32, 99, 114, 117, 118]. They showed that cell extracts from XP-A cells were able to incise UV-irradiated naked DNA as were cell extracts from normal cells, but that the XP-A extracts, unlike the normal extracts, were not able to incise the damaged DNA when it was present in chromatin [117, 118]. These studies utilized two of the XP-A cell lines examined by Lambert et al. [25, 32]. One cell line Lambert used was XP12BE (GM02250) in which UDS was <2% of normal [26]. This patient was a compound heterozygote for two mutations in the *XPA* gene, one in intron 3 and one in exon 4 [26]. The patients of this cell line are considered to have a milder phenotype and intermediate neurological abnormalities) [26, 119]. The second cell line used was XP2OS (GM02345) in which there was a mutation in intron 3 of the *XPA* gene and UDS was <2% of normal [26]. The patients of this cell line were considered to have a severe phenotype [26]. Mortelmans et al in their study utilized three XP-A cell lines: XP12BE, XP12(SV40)RO, and XP25RO, a cell line in which there is a mutation in exon 5 and in which the patients have a severe phenotype [117]. The XP12BE cell line was used in the studies of Lambert et al. Kano and Fujiwara in their study examined two XP-A cell lines: XP2OS and XP6KO [118]. The XP20S cell line was also used in the studies of Lambert et al. Thus, there have been at least five different XP-A cell lines which have produced similar results regarding endonucleolytic activity on UVC-irradiated naked and nucleosomal DNA/chromatin with overlap between investigators on two of these cell lines [26, 117, 118].

In contrast to the studies described above, other studies have shown that in XP-A cells there is decreased repair of damaged naked DNA compared to the repair activity in normal calls [100, 101]. In addition, in normal cells, there are studies which show that there is decreased DNA repair of damaged nucleosomal DNA and chromatin compared to repair of damaged naked DNA [106, 120–125]. These studies included examination of repair of pyrimidine (6-4) pyrimidone photoproducts, cyctobutane pyrimidine dimers, and acetylaminofluorene-guanine (AAF-G) adducts using purified core DNA repair proteins or cell extracts [106, 120–125]. There are a number of factors that could account for some of the differences in the studies mentioned above. These include differences in the presence or absence of chromatin-remodeling factors, in the concentration of salt used in the reaction buffers, or in the XP-A cell lines used. These various possibilities will be discussed in the sections below.

## Effect of chromatin-remodeling proteins on endonucleolytic incision of damaged nucleosomal DNA

One important factor which could be important in accounting for some of the differences observed in endonucleolytic activity on damaged nucleosomal DNA could be due to the presence of chromatin-remodeling proteins. Accessibility of DNA in chromatin can be affected by chromatin-remodeling factors such as chromatin-remodeling proteins and high mobility group (HMG) proteins [121–123]. HMG proteins bind to nucleosomes and can induce changes in histone binding to nucleosomal DNA as well as in the interactions of other proteins with nucleosomal DNA [126–128]. There is not a great deal of knowledge on the influence of HMGs on NER in chromatin, though they have been shown to enhance transcription by inducing an extended conformation in chromatin fibers [126–129]. However, chromatin-remodeling factors have been identified which facilitate DNA repair at various stages in the DNA repair process [121–123, 125]. There are two major groups of chromatin- remodeling complexes: (1) One group of complexes alter DNA-histone interactions through modification of histones by acetylation, phosphorylation, and methylation; and (2) A second group, which is composed of ATP-dependent complexes, uses ATP hydrolysis to locally alter the association of histones with DNA [130–132]. The ATP-dependent complexes are classified into three groups: SW12/SNF2, ISWI and Mi [130–132]. There are several reports which implicate ATP-dependent chromatin-remodeling complexes in recombination and DNA repair. One study found that SWI/SNF increased cleavage of the V(D)J recombination signal sequence in a mononucleosome by RAG1/RAG2 recombinase [125]. Another study has shown that a recombinant *Drosophilia* ACE, a component of the ISWI group of chromatin-remodeling factors, enhanced dual incision activity of combined purified recombinant human NER proteins, RPA, XPA, TFIIH, XPF-ERCC1 and XPG, by 3.8 fold in the linker region of a dinucleosome containing pyrimidine (6-4) pyrimidone photoproducts [106, 123–125]. These studies showed that there was a functional connection between ATP-dependent chromatin-remodeling and NER [106, 123–125].

Studies on chromatin-remodeling proteins and DNA repair have been extended to include additional types of DNA damage. Hara and Sancar using a reconstituted mononucleosomal substrate found that six purified core human NER proteins, RPA, XPA, XPC, TRIIH, XPG and XPF-ERCC1, together had decreased repair activity on UVC and AAF-G damaged mononucleosomes compared to their activity on damaged naked DNA [121, 122]. This deficiency in activity on damaged nucleosomal DNA was reversed and DNA repair activity increased when the SWI/SNF chromatin remodeling factor from yeast was added to the system [122, 123]. The rate and extent of excision of AAF-G adducts from the nucleosome core was stimulated by a factor of about 2 and excision of the (6-4) photoproduct from the linker region, by about 1.5 fold [123]. The repair of thymine dimers in the mononucleosome core particle was not stimulated by SWI/SNF [123]. Other chromatin remodeling factors were proposed to be needed for excision of dimers from the core particle [123]. These studies additionally suggested that XPA, RPA, and XPC are involved in recognition of the damage and recruitment of SWI/SNF to the nucleosome leading to increased DNA accessibility and repair [122, 123]. It was not determined whether one of these three repair proteins or if all three were responsible for stimulating SWI/SNF activity and nucleosomal DNA accessibility [122, 123].

The reconstituted nucleosomal system utilized by Lambert et al., as already mentioned, contained a plasmid with the SV40 and pBR322 genomes, 23 nucleosomes per DNA molecule, and normal human or XP-A lymphoblastoid cell histones [25, 32]. The endonuclease complex used in these studies included the same core proteins as were present in the studies of Hara and Sancar [122, 123]. As has already been mentioned, endonucleolytic incisions produced by this complex increased 2.3-fold on UVC-damaged core nucleosomal DNA, which contained pyrimidine dimers, compared to damaged naked DNA (Figure 6A) [32]. Endonucleolytic activity on this substrate increased as the number of nucleosomes increased, even though the number of pyrimidine dimers on the DNA remained the same [32]. This suggests that the presence of nucleosomes was leading to an increase in the accessibility of sites of damage to endonucleolytic activity.

Since the studies of Hara and Sancar suggest that XPA, RPA and XPC, either singly or in combination, are involved in localizing to sites of nucleosomal DNA damage and in recruitment of and activation of SWI/SNF at these sites, it would be interesting to speculate that XPA, present in the normal endonuclease complexes in the studies of Lambert et al., could play an important role in recruitment of chromatin-remodeling factors, proposed to be present in these complexes, to sites of damage in nucleosomal DNA [25, 32, 122, 123]. This in turn could increase the accessibility of damaged sites and lead to the increase in endonucleolytic activity observed on damaged nucleosomal DNA in these studies. It could also be speculated that increasing the number of nucleosomes on the DNA could first lead to enhanced binding of chromatin-remodeling proteins at the damage site and then these proteins in turn recruit XPA leading to increased accessibility of these sites to endonucleolytic incision This has been suggested as a possibility in some systems [121–123].

It could additionally be speculated that mutations in the *XPA* gene in XP-A cells could lead to decreased association of the mutant XPA protein with chromatin-remodeling factors, which in turn could lead to decreased recruitment of these remodeling factors to nucleosomes, decreased activation of these factors, and decreased accessibility to endonucleolytic incision on the damaged DNA. Alternatively, if as has also been suggested, chromatin remodeling factors first localize to damaged nucleosomal DNA and are activated after they recruit the repair proteins, loss or a defect of a repair protein such as XPA could still lead to decreased activity of the remodeling factor [124]. Thus, interaction of XPA in the normal human endonuclease complexes with chromatin-remodeling proteins, proposed to be present in these complexes, could potentially aid in increasing the accessibility of damaged nucleosomal DNA [25, 32].

## Effect of KCl concentration on a processive versus a distributive mechanism of DNA endonuclease action

A second factor which could possibly explain some of the differences observed in activity of endonucleases in XP-A cells on damaged DNA compared to undamaged DNA, is the concentration of salt used in the buffers in these studies. It is important to keep in mind that when examining proteins which utilize a processive mechanism of action that this mechanism of action is extremely sensitive to salt concentration [87, 133–136]. The reason for this is that a processive mechanism of action involves an electrostatic interaction between a protein and DNA. When the salt concentration is increased, the affinity of a protein for nontarget DNA decreases and a protein with a processive mechanism of action can switch to a distributive mechanism of action [133–137]. Increasing the KCl concentration can also differentially affect the activity of enzymes that utilize a processive versus a distributive mechanism of action [85, 137]. An increase in KCL levels can have an inhibitory effect on enzymes that act by a distributive mechanism but much less of an effect on those that utilize a processive mechanism [85, 137]. The presence of monovalent anions other than chloride, as well as variation in binding and/or compartmentalization of ions such as K^+^ and Na+ can also play a role in electrostatic interactions between proteins and DNA and account, in part, for the differences observed between the various studies carried out [138, 139].

Studies by Lambert et al have shown that when the KCl concentration in the reaction buffer is increased to 70 mM KCl, the endonucleases in the normal endonuclease complex, pI 7.6, switch from a processive to a distributive mechanism of action [32]. The mechanism of action of the XP-A endonucleases remains distributive [32]. The higher KCl concentration also differentially affects the activity of the normal and XP-A endonucleases on damaged naked and nucleosomal DNA [32]. At 70 mM KCl, the number of incisions produced by the normal endonucleases on UVC-irradiated naked DNA increases by approximately 1.4 fold than when the KCl concentration is 0 mM (Figure 6A). In contrast, the number of incisions produced by the XP-A endonucleases decreases and is approximately 3.5 fold less than those produced by the normal endonucleases at 70 mM KCl (Figure 6B). At this KCl concentration, incisions produced by the normal endonucleases on core and total nucleosomal DNA decrease to 60% and 50%, respectively, of those produced on damaged nucleosomal DNA (Figure 6A [32]. In contrast, at 70 mM KCl the XP-A endonucleases produce no incisions on either damaged core or total nucleosomal DNA (Figure 6B) [32]. Similarly, studies on T4 endonuclease V, which uses a processive mechanism to scan DNA containing pyrimidine dimers, indicate that this endonuclease switches to a distributive mechanism of action, when the NaCl concentration is increased to 100 mM [85, 87].

These studies indicate that the salt concentration and ionic strength of the reaction milieu have a major influence in determining the mechanism of action an endonuclease utilizes to localize target sites and whether an endonuclease incises damaged DNA using a processive versus a distributive mechanism of action. This is of particular importance when nucleosomal DNA is being examined and needs to be taken into account when studies are carried out examining the function of proteins which utilize a processive mechanism of action.

## Domains in the XPA protein important for its ability to act as a processivity factor

**FIGURE 8:**
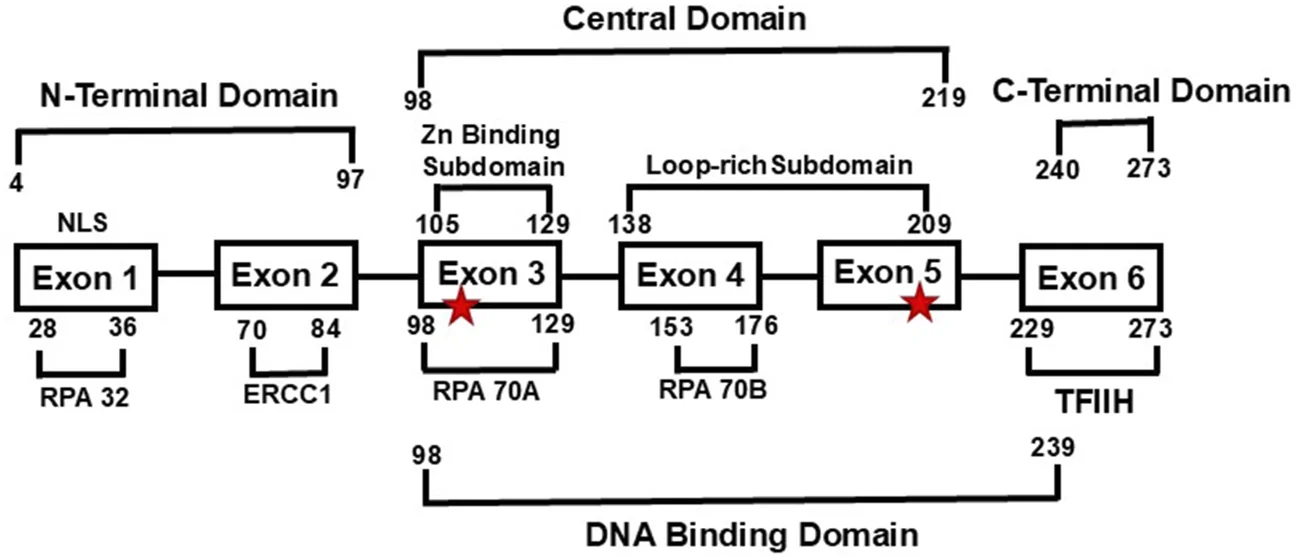
A map of the *XPA* gene and the domains of the XPA protein each encodes. Numbers refer to amino acid number of the XPA protein. The 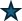 (star) in exon 3 and exon 5 indicates mutations in these exons that lead to expression of XPA proteins that were not able to correct the defect in the ability of XPA to confer a processive mechanism of action on endonucleases involved in NER, and were thus needed for the ability of XPA to act as a processivity factor for these endonucleases (Modified from Bartels and Lambert [33] with permission from Elsevier Inc.).

Studies have been carried out by Lambert et al to examine the domains in the *XPA* gene that are important for the ability of the XPA protein to function as a processivity factor. Using site-directed mutagenesis, mutations were created in several of the exons of the *XPA* gene [33]. The mutant recombinant proteins were examined to determine which mutations eliminated the ability of the XPA protein to confer a processive mechanism of action on the endonucleases in XP-A cells and thus indicate which of the domains were critical for the ability of XPA to act as a processivity factor [33]. Two specific domains were examined. These were the N-terminal domain, which is important in incision activity of the endonucleases involved in NER and the DNA binding domain which is critical for DNA binding (Figure 8) [140–144]. Site-directed mutagenesis studies were carried out using plasmids containing the normal human *XPA* cDNA or mutated *XPA* cDNAs which were then transformed into *E. coli* [33]. Mutations were created in exon 2 (E2), in the N-terminal domain, and in exon 3 (E3) and exon 5 (E5), in the DNA binding domain of the *XPA* gene [33]. The recombinant native XPA protein and the recombinant E2, E3, E5 mutant XPA proteins were extracted, purified and examined for activity on a 93-bp UVC-irradiated DNA substrate which contained cyclobutene pyrimidine dimers [33].

**FIGURE 9:**
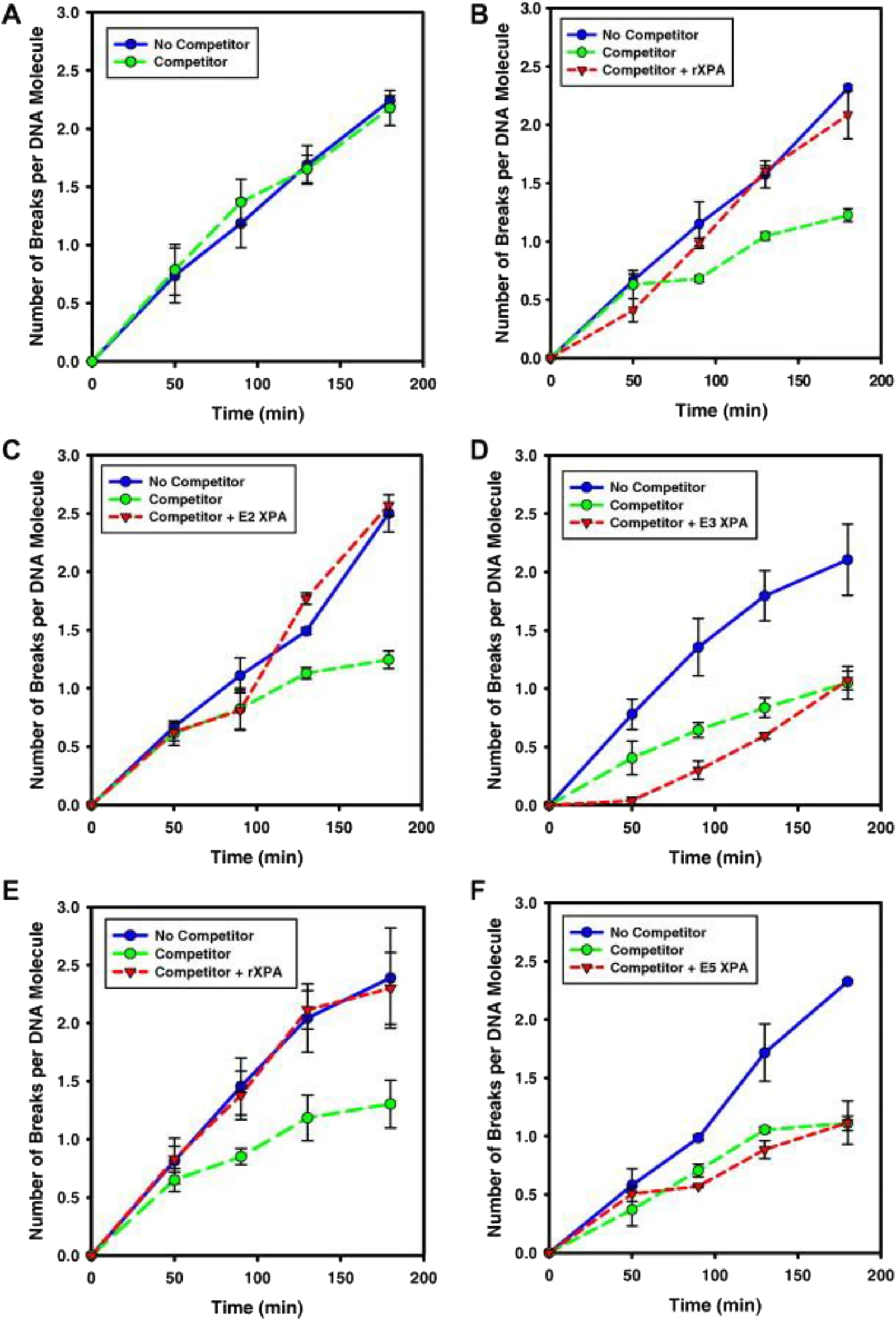
Influence of the native and mutant XPA proteins on the mechanism of action utilized by NER endonucleases in XPA cells for locating sites of damage. Endonuclease activity was measured on UVC-irradiated DNA in the presence of a similarly damaged competitor DNA. **(A)** Chromatin-associated proteins from normal cells were incubated with UVC-irradiated DNA for 40 min after which time the UVC-irradiated competitor DNA was added and incubation continued. **(B–F)** Chromatin-associated proteins from XPA cells plus either native or mutant XPA proteins were incubated with UVC-irradiated DNA for 40 min and then the UVC-irradiated competitor DNA was added and incubation continued. The XPA proteins included in the reactions were: **(B)** recombinant native XPA (rXPA); **(C)** E2 mutant XPA; **(D)** E3 mutant XPA purified only on a Ni^2+^-NTA column; **(E)** rXPA purified only on a Ni^2+^-NtA column; and **(F)** E5 mutant XPA. Endonuclease activity was expressed as the number of breaks per DNA molecule. Vertical lines represent +SEM for three to four experiments (Reproduced from Bartels and Lambert [33] with permission from Elsevier Inc).

A mutation was generated in a region of exon 2 of the *XPA* gene which has been shown to be critical for binding of XPA to ERCC1 and for incision activity in NER [78, 141]. The E2 mutant XPA protein was generated by creating a 12-bp deletion (i.e., deletion of Gly 72-Phe 75) in exon 2 [33]. The E2 mutant XPA protein was able to bind to the UVC-irradiated DNA [33 ]. Whether the E2 mutant XPA protein was able to restore the ability of the endonucleases in the XP-A complex to incise UVC-damaged DNA by a processive mechanism of action was determined. For these studies an assay was used in which endonuclease activity was examined in the presence or absence of a UVC-irradiated competitor DNA (Figure 9A) [33]. Addition of the E2 mutant XPA protein to the assay, which contained chromatin-associated endonucleases from XP-A cells, lead to increased endonuclease activity on the UV-irradiated substrate DNA compared to the competitor DNA (Figure 9C) [33]. The activity of the E2 mutant XPA protein was just like the activity of the recombinant native XPA protein (Figures 9B,E). This indicates that the mutation in the E2 exon of the *XPA* gene did not affect the ability of the XPA protein to stimulate the XP-A endonucleases to locate sites of damage by a processive mechanism of action [33].

Two different mutations were generated in the DNA binding domain of the *XPA* gene in exons 3 and 5 (Figure 8) [33]. XPA patients with more severe symptoms, including central nervous system disorders, have mutations in the DNA binding domain of the *XPA* gene [119, 145]. One of these mutations was in exon 3 in the zinc finger motif of the zinc-binding subdomain, which is critical in NER (Figure 8) [140, 146, 147]. Replacement of any one of the cysteines in the zinc finger with a serine has been shown to disrupt NER and lead to drastic reductions in cellular resistance to UV-irradiation [139, 146]. A mutation in the zinc finger motif in one of the cysteines (Cys 105 to Ser) resulted in the E3 mutant XPA protein [33]. This protein was not able to selectively bind to UVC-irradiated substrate DNA [33]. It was also unable to restore the ability of the XP-A endonucleases to incise the damaged DNA by a processive mechanism of action; it did not increase the number of incisions the XP-A endonucleases produced on UVC-irradiated DNA after the competitor DNA was added (Figure 9D) [33]. This indicated that the zinc-finger subdomain of XPA is important for the ability of XPA to stimulate the endonucleases in the XP-A complex to utilize a processive mechanism of action to produce incisions at sites of damage [33].

The second mutation generated in the DNA binding domain was in exon 5 in the loop-rich subdomain in the DNA-binding domain of the *XPA* gene (Figure 8) [142–144]. This subdomain consists of a sheet-helix-loop region and a helix-turn-helix region, between which is a large basic cleft that is very important in DNA binding [143, 144]. The mutation in exon 5 (i.e., Arg 207─>stop) is one that is present in two different XPA patients (XP12RO and XP25RO) who have severe symptoms of XP [26]. The mutation in exon 5 (E5) affects the positive charge in the large basic cleft in this subdomain of XPA and leads to protein termination [143, 144]. The exon 5 mutant XPA protein was not able to bind to UVC-irradiated DNA [25]. It could also not restore the ability of the XP-A endonucleases to incise the damaged DNA by a processive mechanism of action [33]. The E5 mutant XPA protein did not lead to an increase in the number of incisions produced by the XP-A endonucleases on UVC-irradiated substrate DNA after the competitor DNA was added (Figure 9F). These studies indicate that two subdomains of the XPA binding domain, the zinc binding subdomain in exon 3 and the loop-rich subdomain in exon 5, are important for the ability of XPA to bind to damaged DNA and to aid in the processive mechanism of action utilized by the XP-A endonucleases for incision of UV-damaged DNA [25]. Whether there are other regions in the *XPA* gene which are important for this still needs to be examined.

As has been previously discussed, a number of proteins act as processivity factors and can confer a processive mechanism of action on a specific protein [82–84, 148]. A number of these proteins have a cleft, or well-developed groove, that binds to or associates with DNA and allows the protein to translocate along the DNA in association with its specific protein(s) in a processive manner [83, 149]. NMR studies of the basic cleft in the loop-rich subdomain of the XPA binding domain indicate that it could accommodate the phosphodiester backbone of double-stranded DNA [143, 144]. It is thus possible that the structure of the basic cleft in XPA would enable it to track along the DNA in conjunction with other NER proteins, such as XPA and XPG, in search of sites of damage. If there were mutations in this region of the *XPA* gene, as are found in XP-A patients, then the ability of the XPA to act as a processivity factor enabling the endonucleases to scan the DNA and incise at multiple sites of damage could be curtailed, as is proposed for the E5 mutant XPA protein. This would significantly affect the DNA repair process.

Examination of a number of the mutations found the *XPA* gene as well as a number of other XP genes has demonstrated that there is a correlation between the location of the mutation on the gene (i.e. in which exon the mutation occurs), the DNA repair capacity of the specific XP protein encoded by that gene, and the clinical severity of the disorder [26, 119, 145]. Studies are ongoing in examining the specific mutation a XP patient has in the XP gene which is defective and correlating it with the clinical manifestations of the disorder in the patient. These are important studies which should aid in further development of therapeutic approaches for treatment of this disorder.

## Proposed model for interaction of repair proteins with a processive mechanism of action with chromatin-remodeling factors on damaged nucleosomal DNA

Studies indicate that repair proteins utilizing a processive mechanism of action and chromatin-remodeling factors are both important in NER in DNA, particularly on damaged nucleosomal DNA [25, 32, 33, 122, 123, 125, 126]. It has been proposed that XPA acts as a processivity factor, mechanistically aiding in enhancing the processive mechanism of action of several proteins in their role in NER. These include the endonucleases, XPF and XPG, and the XPB/core TFIIH translocase [25, 30–32]. Studies indicate that XPA, RPA and XPC, either singly or in combination, interact with the chromatin-remodeling protein, SWI/SNF, which leads to enhanced accessibility of nucleosomal DNA after damage [122, 123]. A model is now proposed on the interaction of XPA and chromatin-remodeling factors in the repair of damaged nucleosomal DNA.

In this model, after DNA damage and the initial damage recognition step in NER involving XPC, XPA is recruited to the nucleosomes. XPA will be used here as an example of the NER protein recruited. There are two mechanisms which have been proposed for this step. In one, XPA, after recruitment to the damaged nucleosome, recruits a chromatin-remodeling protein to the nucleosome [122, 123]. In the other mechanism, after localization of the chromatin-remodeling protein to the damaged nucleosome, it recruits XPA [122, 123]. A model will be described here in which the XPA protein recruits the remodeling protein. The chromatin-remodeling protein recruited may depend upon the type of DNA damage and its location on the nucleosomal DNA (i.e., core or linker) as has been previously described [122, 123, 125].

After recruitment of the remodeling protein by XPA, the remodeling activity of this protein is activated and the accessibility of sites of damage on DNA is enhanced. XPA mechanistically stimulates XPF and XPG to processively translocate along the damaged nucleosomal DNA, which is now more accessible. The endonucleases processively scan the DNA and remain associated with the DNA until they reach the site of damage. Their ability to scan for damage sites and remain associated with the DNA until these sites are reached increases the efficiency of the process. Dual incisions are produced in the DNA at the site of the lesion. Other NER proteins will be then be recruited to carry out the subsequent steps in the repair process. The endonuclease can then continue to processively scan the DNA for other damage sites. Thus both the action of XPA on recruiting and stimulating the chromatin-remodeling factor(s) leading to increased accessibility of sites of damage on the nucleosomal DNA and the ability of XPA to then stimulate the processive scanning of nucleosomal DNA by XPF and XPG are proposed to be needed for increased endonucleolytic incision of damaged nucleosomal DNA, as has been observed [25, 32, 122, 123, 125].

There is evidence to support this model. Studies have shown that when the normal DNA endonucleases involved in NER are switched from a processive to a distributive mechanism of action, they are unable to incise damaged nucleosomal DNA [32]. In XP-A cells, studies indicate that endonucleases use a distributive mechanism of action and are unable to incise damaged nuclesomal DNA [25, 32]. The deficiency in ability of the endonucleases to act processively in XP-A cells can be corrected by a recombinant XPA protein [25]. Studies using a reconstituted mononucleosome indicate that XPA, RPA and XPC, either separately or together, and SWI/SNF are both needed for incision of damaged nucleosomal DNA [122, 123]. Collectively, these studies suggest that chromatin-remodeling factors and XPA are critical for enhanced endonuclease activity on damaged nucleosomal DNA. This model thus proposes that XPA has two important functions: it acts as a processivity factor enhancing the processivity of endonucleases involved in NER allowing a more efficient progression of the repair process, and it recruits and stimulates chromatin-remodeling factors to enhance accessibility of damage sites on nucleosomal DNA. It is possible that this same model could be applied to the interaction of XPA with XPB/core TFIIH. This protein interaction needs to be examined in damaged nucleosomes in both normal human and XP-A cells. Additionally, it could be hypothesized that defects in the ability of XPA to enhance a processive mechanism of action of the endonucleases in NER and to potentially recruit chromatin remodeling proteins to damaged nucleosomal DNA could be an important factor in the clinical manifestations of the disorder observed in XP-A patients.

## Strategies for therapeutic intervention in XP

As has been noted, exposure of XP patients to ultraviolet light can lead to skin dyspigmentation, freckling, and xerosis (dryness) at a very early age. This can be followed by development of sun-induced neoplasms which include actinic keratoses, squamous cell carcinomas, basal cell carcinomas and melanomas, often in large numbers [2–9, 22–24]. These changes are due to mutations induced in genes in the skin keratinocytes and melanocytes, and in genes which regulate the cell cycle in these cells and in cells in underlying tissues [3, 10, 22–24]. XP patients are markedly heterogeneous in the sun-induced changes and development of the clinical manifestations seen in this disorder, with differences occurring both between and within XP complementation groups. Treatment regimens for patients have thus had to be developed for each individual patient based on the particular changes noted in that patient and on his or her response to therapy.

Since XP patients are extremely sensitive to UV-irradiation induced lesions in DNA and are defective in either repair of these lesions by NER (XP complementation groups XP-A through XP-J) or ability to carry out DNA replication past a site of damage by translesion DNA synthesis, (XP variant, XP-V), it is extremely important to understand the genes involved in these processes and the mechanism of action of the proteins encoded by these genes. Extensive research has been undertaken and is still ongoing, particularly in development of potential NER-targeted chemotherapeutics. Understanding the mechanism of action of the protein machinery involved in NER or translesion DNA synthesis is extremely important for development of therapeutic methodologies to treat patients with this disorder.

Over the years, a number of protocols have been developed for treatment of XP patients, particularly regarding removal of malignancies which can develop in these patients and can be quite numerous. These include: excision of the tumor, which can be effective, if excised before metastasis. However, due to the extensive number of cancers which can form there is limited ability of affected skin tissue to tolerate this. Other methods such as the use of oral retinoids (isotretinoin) have been effective in a number of cases, however, the toxicity of these reagents has limited their use [20, 24].

Importantly, methodologies have been developed and are currently being worked on to prevent occurrence of these malignancies in the skin. Knowledge of the genes and proteins involved in the NER process and the specific effect mutations in the repair genes have on the clinical manifestation of the disorder in these patients is aiding in this process. Several protocols that are in use and have been successful in a number of XP patients are described below. A great deal more methodologies need to be developed.

### Cancer prophylaxis in XP patients with topical 5-fluorouracil

Treatment of XP patients with topical 5-fluorouracil (5-FU) has been found to be effective in prevention of development of skin cancers in these patients [23]. 5-FU is an antimetabolite drug which has been used as first-line chemotherapy to treat a number of different types of cancer such as head, neck, breast and colorectal cancer [150–153]. When cells are treated with 5-FU, this leads to inhibition of thymidylate synthase and to increases in dUTP and 5-F-dUTP and their incorporation into genomic DNA [152, 154]. This results in extensive problems in progression of the DNA replication fork, causing accumulation of cells in S-phase [152, 154–156].

In XP patients, topical treatment of sun exposed areas of the skin with 5-FU (e.g. Effudex®) has been successful in prevention of cutaneous malignancies that can arise in these patients [23]. For example, application of 5-FU at sites of evident or subclinical actinic keratoses, which can develop into baso-squamous carcinomas, has been shown to lead to clearance of the lesions [23]. It is hypothesized that the blockage of DNA synthesis and cell replication that occurs in cells in the skin after exposure to 5-FU allows the cells time to carry out DNA repair. Even though cells in XP patients have mutations in specific XP genes and defective XP proteins, some DNA repair still goes on in these cells. The level of DNA repair depends upon the complementation group (Table 1). It can be hypothesized that the low levels of DNA repair ongoing in the XP cells, which differ with the complementation group, may be sufficient to allow enough repair to take place in these slower dividing cells treated with 5-FU so that it may aid in preventing cancer development. Studies indicate that use of 5-FU can aid in prevention of skin and mucous membrane cancer in sun-exposed sites in XP patients, though treatment needs to be adjusted based on the individual [23]. Some but not all XP patients treated with 5-FU develop inflammation in developing skin tumors, a process known as pytaptosis. Beginning treatment in childhood minimizes this problem.

### Prophylactic treatment of XP patients with topical imiquimod

Another topical agent used in the treatment of XP patients is imiquimod (e.g. Aldara®) [23, 157–160]. Imiquimod is an immune response modifier which has an anti-proliferative effect against developing skin cancers [157–160]. It also has a separate activity which stimulates the inhibitory pathway which regulates cell proliferation by modulating cyclin dependent kinase inhibitors, resulting in retarding cells at the G_1_-S interface of the cell cycle [161]. Thus, like 5-FU, it may slow down cell division enough to allow the limited DNA repair capabilities of the XP cells to repair some of the UV damaged DNA. Imiquimod, like 5-FU, has been shown, when applied to sun-exposed areas of the skin, to prevent skin cancer development [23].

It has been recommended that XP patients should be started on treatment with 5-FU and imiquimod in early childhood, as soon as possible following the diagnosis of XP and the appearance of skin stigmata of XP such as dyspigmentation and xerosis and when neoplasms are just beginning to develop [23]. These prophylactic agents can be used on a systemic basis and are based on the premise that they can block DNA synthesis and cell replication thus aiding in some limited DNA repair to take place [23]. As for all XP patients, those who are receiving various prophylactic treatments need to continue to exert extreme care to stay out of the sun, to use sunscreens and protective clothing when exposed to sunlight and to make sure windows in the home and car have protective sun films.

### Treatment of XP patients by introduction of T4 endonuclease V in liposomes into XP skin cells

Another method that has been developed for lowering the rate of formation of new skin cancers in XP patients utilizes a bacteriophage DNA repair protein. This method was developed by Yarosh et al [21, 162, 163]. In these studies, a bacteriophage DNA repair protein, T4 endonuclease V, was encapsulated into an engineered pH-sensitive liposome for delivery into the cells of the skin [21, 162, 163]. T4 endonuclease V primarily incises DNA at sites of pyrimidine dimers leading to removal of the lesion [164, 165]. The liposomes containing T4 endonuclease V were then topically applied in a hydrogel lotion (T4N5) to the skin of XP patients [21, 163]. It was found that this method of treatment accelerated the removal of UV-induced DNA damage, primarily pyrimidine dimers, with no major adverse effects. It was found that the T4N5 liposome lotion reduced the rate of appearance of new actinic keratoses and their development into basal-cell carcinomas in XP patients, with the greatest success found in patients under 18 years of age [21, 163]. No XP variant patients were included in this study. The efficacy of the treatment was still present during the 6 months following cessation of the treatment. The T4 endonuclease V delivered by liposomes was localized in the epidermis and did not readily penetrate into the dermis which may explain the absence of toxicity of the T4N5 lotion [21, 163]. It was expected that continued use of this T4N5 lotion would be needed throughout life. T4 endonuclease V, however, was only effective on repair of pyrimidine dimers present in the epidermal cellular DNA. It did not reverse mutations that had already occurred in any of these cells.

This liposome encapsulation of T4 endonuclease V represented a new drug delivery approach that introduced a DNA repair protein into epidermal cells which could repair DNA damage produced by UV-irradiation to the skin, was safe and had low toxicity [21, 162, 163]. The clinical trials for this method were very successful and promising and the phase III study of T4N5 liposomal topical lotion was FDA approved. Studies on this treatment have not progressed further. These studies demonstrate, however, the development of a method in which UV-induced DNA damage in the skin can be repaired by introduction of a specific DNA repair protein into cells in the skin via liposome mediated transport. Though this method provides temporary correction of the repair defect, if continued it could potentially greatly enhance the life expectancy of these XP patients. Knowledge of the effect a specific mutation in an XP gene could have on the functioning of a particular XP protein could aid in development of similar technologies to introduce specific repair proteins into epidermal cells in the skin of XP patients.

Effective therapeutic interventions for XP are needed. In some genetic disorders approaches acting at the promotor, RNA, or protein level using small-activating RNA (saRNA), small interfering RNA (siRNA) or allosteric modulators are being investigated [166]. In XP-J, antisense oligonucleotides (ASOs) are being examined as a platform for tailored therapies and could allow modulation of gene expression parameters [12, 166]. This method is being examined to stabilize the TFIIH complex [12]. It has been proposed that ASO-mediated interventions may be a step towards development of specific therapies of patients with XP [166].

## Discussion

### Summary and perspective

Xeroderma pigmentosum (XP) is a hereditary disorder in which there are deficiencies in DNA repair and DNA replication. XP involves eight genes (*XPA* through *XPG* and *XPJ*), which are components of the nucleotide excision repair (NER) system, and an XP variant gene (*POLH*) which is important in lesion bypass during DNA replication. Importantly, studies have been and are currently being carried out to relate specific mutations in these genes with changes in the behavior and function of the encoded NER proteins and with the clinical manifestations of the disorder. Critical for these studies is elucidation of the molecular mechanisms utilized by these repair proteins and the changes that occur in them due to mutations in the XP genes.

This review has focused on one XP complementation group, XP-A. A large number of patients in the XP-A complementation group are among the most severely affected and have the lowest levels of DNA repair, which is 2–5% of normal levels. The *XPA* gene codes for the XPA protein which plays a central role in NER. The XPA protein acts as a scaffold and brings about distinct protein-protein interactions between key components of NER and is critical for guiding the positioning of these proteins to the damage site.

XPA plays an important role in stimulating the ability of endonucleases involved in NER (XPF and XPG) to scan and localize to sites of UV-damage using a processive mechanism of action, which has been shown to significantly increase the rate and efficiency of location of target sites. Studies also indicate that XPA increases the processivity of the XPB/core TFIIH complex as it translocates along DNA enhancing the unwinding of the DNA repair bubble during NER. Thus, XPA is proposed to act as a processivity factor for two different groups of proteins involved in NER, the endonucleases, XPF and XPG, and the helicases, XPB/core TFIIH.

The action of XPA as a processivity factor has also been suggested to be critical for endonucleolytic incision of UV-damaged nucleosomal DNA. Studies have shown that when normal human DNA endonucleases switch from a processive to a distributive mechanism of action they loose their ability to incise damaged nucleosomal DNA. In XP-A cells, from several XP-A cell lines, endonucleases incise DNA utilizing a distrubutive mechanism of action and are defective in ability to incise damaged nucleosomal DNA. The importance of XPA in enabling the endonucleases to act processively is seen when a recombinant XPA protein is expressed in these XP-A cells and this leads to the endonucleases utilizing to a processive mechanism of action and their ability to incise damaged nucleosomal DNA. Studies have shown that production of mutations in exons 3 and 5 in the *XPA* gene, which are in the DNA binding domain of *XPA*, lead to loss of ability of XPA to act as a processivity factor. The mutation produced in exon 5 is found in two XP-A patients with very severe forms of the disorder. This correlates with findings that mutations in the DNA binding domain of *XPA* produce more severe CNS disorders. Whether there are other mutations in the *XPA* gene which produce a similar effect on the XPA protein needs to be further examined. These studies indicate that there is a correlation between a mutation in the DNA binding domain of the *XPA* gene, a deficiency in a particular function of the XPA protein (i.e. its ability to function as a processivity factor) and the severity of the disorder.

Another area of particular interest is the role of chromatin-remodeling factors in NER and the interaction between these remodeling factors and several core NER proteins. In a reconstituted nucleosomal system utilized by Lambert et al, endonucleases in chromatin-associated endonuclease complexes were shown to have over two-fold increased activity on UV-damaged nucleosomal DNA. This is in contrast to studies in which no increase in endonuclease activity was seen when various cell extracts or purified proteins were used. Studies by Hara and Sancar using a reconstituted mononucleosome as substrate indicate that XPA, RPA and XPC, either singly or in combination, recruit the chromatin remodeling factor SWI/SNF to nucleosomes and increase endonucleolytic incision on damaged UV- or AAF-damaged nucleosomal DNA. Based on these combined studies it is hypothesized that XPA can recruit chromatin-remodeling factors to damaged nucleosomal DNA, which increases the accessibility of nucleosomal DNA to endonucleolytic incision. The ability of XPA, in turn, to enhance the processivity of endonucleases in NER leads to increased incisions on the damaged nucleosomal DNA. Thus, XPA is hypothesized to be involved in both the recruitment of chromatin remodeling factors to nucleosomes to increase accessibility of sites of damage and to enhancing a processive mechanism of action of DNA endonucleases, which aids in incision of damaged nucleosomal DNA. Both of these processes are critical for NER repair of nucleosomal DNA.

There are treatments available for XP, some have been more successful than others. Agents such as 5-fluorouracil and imiquimod have been applied topically to the skin of XP patients and their effectiveness, at least in part, results from them having an antiproliferative effect against developing skin cancers, especially when applied at an early stage. It is thought to be in part the result of slowing down cell division enough to allow limited DNA repair capabilities to take place in the XP cells. Another promising treatment has been seen in early clinical trials using a bacteriophage DNA repair protein, T4 endonuclease V, which incises DNA at sites of pyrimidine dimers. Delivery of T4 endonuclease V, encapsulated into a liposome, to the skin resulted in removal of UV-induced DNA damage with no major adverse effects and reduced the rate of development of basal-cell carcinomas in XP patients. Though the clinical trials were very successful and promising, they have not progressed further. These studies, however, suggest a novel method for introduction of specific repair proteins into the epidermal cells in the skin of XP patients. There is a need for development of more clinical trials such as this one.

There are a number of unanswered questions in the studies mentioned in this review. Regarding the proposed role of XPA in enhancing the processivity of several of the proteins involved in NER and a deficiency in this ability in several XP-A cell lines, it will be important to determine whether this deficiency is present in a number of other XP-A cell lines and whether other mutations in the *XPA* gene may be involved in this, in addition to those which have already been examined. Studies on XPA’s role in enhancing the processivity of XPB/core TFIIH translocase activity on damaged DNA needs to be carried out using human cells and also XP-A cells. It would be of interest to determine whether in XP-A cells there is a deficiency in translocation of XPB/core TFIIH along the damaged DNA and the effect that nucleosome structure would have on this process. In addition, examination of the interaction of XPA with chromatin-remodeling proteins needs to be extended to examine the effects of remodeling factors on damaged nucleosomal DNA in XP-A cells and comparing the results of these studies with those using normal cells. A reconstituted nucleosomal system containing damaged DNA and NER proteins from normal and XP-A cells could be used since these types of systems have provided important information in the past. Studies of this nature would answer critical questions regarding the importance of a processive mechanism of action of specific NER proteins in the repair process on damaged nucleosomal DNA, and the importance of the interaction of these proteins with chromatin-remodeling factors on damaged nucleosomal DNA. It would be interesting to determine if XPA effects the processivity of other proteins in NER and in doing so may be involved in additional types of interactions with proteins in the NER process. If any of these interactions involve damaged nucleosomal DNA and chromatin-remodeling proteins, they could be important at a number of different levels of DNA repair. The ultimate goal will be connecting the underlying mutations in the XP-A gene with the different pathophysiological changes observed at the cellular level and with the clinical manifestations of XP and then correcting these underlying mutations.
